# Synchronizing Nitrogen Fertilization and Planting Date to Improve Resource Use Efficiency, Productivity, and Profitability of Upland Rice

**DOI:** 10.3389/fpls.2022.895811

**Published:** 2022-05-18

**Authors:** Tajamul Hussain, Hero T. Gollany, Nurda Hussain, Mukhtar Ahmed, Muhammad Tahir, Saowapa Duangpan

**Affiliations:** ^1^Laboratory of Plant Breeding and Climate Resilient Agriculture, Agricultural Innovation and Management Division, Faculty of Natural Resources, Prince of Songkla University, Hat Yai, Thailand; ^2^United States Department of Agriculture, Agricultural Research Service (USDA-ARS), Columbia Plateau Conservation Research Center, Pendleton, OR, United States; ^3^Department of Agronomy, Faculty of Crop and Food Sciences, Pir Mehr Ali Shah (PMAS)-Arid Agriculture University, Rawalpindi, Pakistan; ^4^Department of Soil, Water, and Climate, University of Minnesota, Falcon Heights, MN, United States

**Keywords:** agronomic management, nitrogen uptake, recovery efficiency, crop water productivity, grain yield

## Abstract

Synchronizing nitrogen (N) fertilization with planting date (PD) could enhance resource use efficiency and profitability of upland rice (*Oryza sativa* L.) production in Thailand. The objective of the study was to assess upland rice responses to four N fertilization rates (NFRs) and three planting dates. Field experiments were conducted during two growing seasons under four NFRs, no N applied (N_0_), 30 (N_30_), 60 (N_60_), and 90 kg N ha^−1^ (N_90_), and NFR were applied at the initiation of tillering and panicle emergence stages. The planting dates selected were early (PD1), intermedium (PD2), and late planting (PD3) between September and December of each season. The NFRs and planting dates had a significant influence on N uptake, N use efficiency (NUE), crop water productivity, yield and yield attributes, and profitability of upland rice production. A linear relationship among NFRs, agronomic traits of upland rice, N uptake, and crop water productivity was observed, and a significant seasonal effect was indicated. Fertilization at N_90_ under PD2 enhanced yields, yield attributes, and grain yields, as well as crop water productivity by 56 and 105% during the second and first seasons, respectively. Grain N, total N, and straw N were increased by 159, 159, and 160%, and by 90, 114, and 153%, during the first and second seasons, respectively. Enhanced N efficiencies, including agronomic efficiency, recovery efficiency, partial factor productivity, and N harvest index, at varying NFRs were observed under PD2 during both seasons. Highly significant (*p* < 0.001) and positive associations were observed among agronomic attributes, N uptake, NUE, and crop water productivity of upland rice in correlation assessment. Profitability from grain yields was observed with N fertilization and N_90_ resulted in maximum profit under all the PDs. However, the highest marginal benefit-cost ratio was observed at N_60_ under PD2 during both seasons. The results suggest that the NFR of 90 kg N ha^−1^ and planting at the end of September or start of October would enhance resource use efficiency and productivity, and maximize profitability. Furthermore, long–term field investigations with a range of NFRs and adopting forecasting measures to adjust the planting date for upland rice are recommended.

## Introduction

Rice is a major cereal crop, is a staple food, and is a source of calories, protein, and nutrients; it significantly contributes to the dietary needs in Thailand. Globally, rice is grown in more than 95 countries (IRRI, 2002). Thailand is the sixth major rice-producing country worldwide and is ranked second in Southeast Asia (FAO, 2020). Upland rice contributes 11% of the world's rice production (Jaruchai et al., 2018), 9% of the total rice production area in Asia (Nascente et al., 2019) and Thailand. It is an important crop, contributing to local food security and economy of upland areas. It is also beneficial, as it is grown under rainfed conditions, and additional irrigation water is seldom applied (Kumar and Ladha, 2011). However, grain production of upland rice is low especially in Thailand because of various factors, including seasonal weather patterns and traditional agronomic management practices.

Upland rice is farmed by small landholders in northern and southern regions of the country during the rainy season (Nokkoul and Wichitparp, 2013). The production potential of upland rice is not yet explored because of its limited production in less fertile soils and drought-prone areas. Upland rice is cultivated on upland soils, foothill plains, and slopy and mountainous areas in Thailand. Northern Thailand consists of high mountains where upland rice is cultivated in highlands and steep river valleys. Swidden agriculture is also practiced in northern Thailand where upland rice is grown in rotation with slashing vegetation, tree regeneration, and shift in cultivation (Champrasert et al., 2020). Comparatively low yield production (0.6–0.9 t.ha^−1^) of upland rice has been reported in northern Thailand (Karladee et al., 2012). In the southern region, upland rice is cultivated as a sole crop or is intercropped with young rubber, oil palm, and other fruit trees, and is found to be the most favorable crop for intercropping with young rubber, oil palm, and other trees. Because of its feasibility for intercropping, experiments conducted in Songkhla province confirmed that upland rice is a potential option to meet grain needs and is not affecting the young rubber if sufficient fertilizer is applied. Furthermore, a coordinated program between the Rubber Department and Rice Department of Thailand identified two local cultivars, Dawk Pa-yawm and Kho Muang Luang, as the most suitable cultivars for intercropping with young rubber and are recommended for general cultivation (Laosuwan, 1996), which increased the production of upland rice in southern Thailand. However, no further study has been conducted to analyze the impact of seasonal variations in weather patterns on upland rice response to fertilizer recommendations, which have led to continuous traditional agronomic management practices resulting in declined productivity in Thailand.

Stable upland rice production is a significant factor to meet increasing demand and ensuring food security. Climate change has affected rice production because of changes in seasonal variability in rainfall and increases in average temperature. In this scenario, maintaining a higher yield per unit area is a primary objective of upland rice production systems. In comparison to climatic factors, including air temperature, rainfall, solar radiation, soil moisture, and insects, pests, and weeds, planting time (Ferrari et al., 2018) and nitrogen (N) fertilization management are factors that are highly associated with yields and are easy for farmers to adjust and manipulate. Nitrogen is a critical nutrient that affects crop growth (Hameed et al., 2019; Santiago-Arenas et al., 2021), hence, significantly influencing crop productivity. Nitrogen deficiency in rice plants causes yellowing of leaves, reduces leaf size, and leads to low productivity, whereas excessive N fertilization results in agronomic and economic losses. Therefore, it becomes imperative that a sufficient and optimum N dose should be applied to obtain stable grain production. In northern areas of Thailand, the application of 10–75 kg N ha^−1^ by farmers in upland rice fields was reported in a survey conducted by Chiang Mai University, Thailand (CARSR, 2003). Different NFRs have been observed as N fertilization of 61.25 kg N ha^−1^ (Suwanasa et al., 2018), 61.25 kg N ha^−1^ (Hussain et al., 2018a,b), and a basal fertilization of 15 kg N ha^−1^(Islam et al., 2020) in upland rice farming in southern Thailand. Corresponding to the Division of Rice Research and Development (DRRD) of Thailand (DRRD, 2016; Norsuwan et al., 2020), 48.75–82.5 kg N ha^−1^ based on soil N status was recommended to be used as N fertilization management for rice production. In addition to this, DRRD advised split application of 40–45 kg N ha^−1^, including 20–45 kg N ha^−1^ as basal dose and remaining dose before heading stage for foothill rice areas (DRRD, 2017). Fertilization of 34–39 kg N ha^−1^ for photoperiod–sensitive and 59–69 kg N ha^−1^ for photoperiod–insensitive was recommended based on photoperiod sensitivity of rice cultivars in Songkhla province (experimental area) of Thailand. Variable ranges of N fertilization prevail in Thailand, and no specific or optimum recommendations have been observed according to different planting times for upland rice production. Therefore, farmers usually practiced fertilization of 10–75 kg N ha^−1^ in upland rice fields. Farmers usually use urea to meet N fertilizer demand. Application of improper dose of urea, which is extremely volatile, results in higher N losses as urea–NUE is lower around 30–40%, in rice production systems, and seldom exceeds 50% (Choudhury and Khanif, 2006). Efficient fertilizer use is also a key component to increase N uptake by rice plants. However, traditional, and inadequate N fertilization practices along with variability in cultivar's efficiency to take up N from soil, existing climatic conditions, including temperature and soil water contents, have affected effective N use. Nitrogen uptake has a direct relationship with NFR. In contrast, higher NFRs result in higher N losses (Zhang et al., 2018) because of increased soil N in the root zone (Belder, 2005). Excessive N use in upland rice may not increase yield as has been observed in numerous studies. Singh and Singh (1976) reported that the upland rice cultivar Bala was responsive up to 90 kg N ha^−1^. Therefore, optimal nutrient management is necessary (Manzoor et al., 2006) to reduce agronomic and economic losses triggered by reduced or excessive N fertilization. To enhance N use efficiency (NUE) and identification of suitable N fertilizer rate (NFR), estimation of plant nutrient concentrations and N uptake are useful indicators. NUE has also indicated a decreasing trend with an increase in N fertilization rates (Barbieri et al., 2008) due to high levels of N in the soil, and NUE in rice production systems has decreased (Santiago-Arenas et al., 2021). Higher NUE achieved at a certain NFR could be used to identify a suitable NFR for upland rice production.

Ideal PD is a useful agronomic management factor for upland rice, and can ensure maximum use of climatic contributors (i.e., photosynthetic radiation, favorable temperature, and precipitation). Planting date affect rice productivity, as soil water status and environmental conditions differ over time. Upland rice is grown during the rainy season in Thailand (Hussain et al., 2018b), and the rainy season lasts from May to October (Limsakul and Singhruck, 2016; Ullah et al., 2019). High variability prevails in the climate of Thailand, and most rain in the east of southern Thailand occurs from November to February of the subsequent year (Limsakul and Singhruck, 2016). Farmers in Thailand perform early or delayed upland rice planting depending on soil water availability. Upland rice planted too early or late is affected by hot and dry intervals when the rice is at the reproductive stages. Too early or delayed planting results in high plant sterility, and the number of effective tillers is reduced (Nazir, 1994). Grain production is also decreased because of the incomplete development of yield-contributing traits at different crop growth phases. The yield potential of a cultivar depends on tillering that occurs at vegetative stages and panicle density that is achieved at panicle formation stages. Unsuitable planting dates and less precipitation at the reproductive stage of upland rice results in higher yield losses (Hussain et al., 2018b). Planting photosensitive upland cultivars in southern Thailand (Watcharin et al., 2020) is another critical aspect affecting upland rice productivity. The recommendation for development and cultivation of photoperiod-insensitive upland rice cultivars to stabilize rice productivity (Watcharin et al., 2020) is also threatened because of the impact of climate change, as climate change has resulted in high rainfall variability and increased drought occurrence (Ullah et al., 2019; Mansour et al., 2021). In this scenario, photoperiod-insensitive cultivars will also be affected because of seasonal variations in rainfall, which cause drought or flood incidents leading to reduced N availability or removal of N from soil surface during high rainfall events. Farmers apply supplementary irrigation to upland rice during hot and dry intervals which increases input cost, and crop water productivity is also affected.

A significant synergy between optimum fertilization, particularly N, and soil water contents is reported in other studies (Santiago-Arenas et al., 2021), which positively influences rice productivity. Adjustment in PD can shift crop period to most favorable period and enable a crop to utilize enough soil water, thus increasing crop water productivity. There is evidence for enhanced NUE with increased crop water productivity under various N applications, and NFR-altered crop water productivity and water input determined NUE (Gajri et al., 1993). Ideal PD is also as important, as it ensures maximum vegetative growth, adjusts the sensitivity of cultivars to difference in temperatures, and enhances grain filling (Farrell et al., 2003). In addition, adjusting the crop growth period according to a suitable PD (Ullah et al., 2019) helps in shifting critical crop growth and developmental phases to the most promising part of the season that ensures maximum use of input resources. Therefore, to reduce and cope with the influence of climate change on upland rice production, proper plant nutrient management and adjustment in PD is essential (Babel et al., 2011; Boonwichai et al., 2019, 2021).

Traditional agronomic practices for N fertilization, general recommendation rates, and prevalence of wide planting windows have led to increased vulnerability of upland rice production. To the best of our knowledge, field evaluations for identifying a suitable NFR alone or synchronized with ideal PDs have not been conducted for upland rice production in Thailand. Therefore, the research was conducted to determine upland rice responses to NFRs and PDs under field conditions. We hypothesized that adjusting PD and application of suitable N rate synchronized with PD assures improved resource use efficiency, enhances productivity, and maximizes profitability of upland rice production.

## Materials and Methods

### Study Site Description

A 2-year experiment was established in the experimental field area of the Faculty of Natural Resources, Prince of Songkla University, Hat Yai, Thailand (7°00'14.5” N, 100°30'14.7” E) during rice growing periods in the 2018–2019 and 2019–2020 crop years. The experimental area is in Songkhla province in the east of Southern Thailand (Supplementary Figure S1). The climate of Songkhla is characterized by a hot or dry season (January–May) and a rainy season (June–December). High variability prevails in the climate of Southern Thailand. Maximum precipitation occurs from November to February of next year in the eastern part of Southern Thailand (Limsakul and Singhruck, 2016). The mean minimum and maximum temperatures reach 24.8 and 31.5°C, respectively, with an annual average temperature of 27.9°C and average annual rainfall of 2,066.7 mm (Hussain et al., 2021a; TMD, 2021). The soil at the study area is well–drained sandy clay loam at the 0–30 cm soil depth. Field capacity, permanent wilting point, and available water capacity of the 0- to 30-cm soil layer are 15.06, 9.44, and 5.62%, respectively. Soil chemical properties of pre-plantation soil analysis include pH, organic matter (Walkley and Black, 1934), total N (Kjeldahl, 1883), available phosphorus (Bray and Kurtz, 1945), and available potassium (Thomas, 1982), and are reported in Supplementary Table S1.

### Treatments and Experimental Setup

The experimental trials consisted of two treatments, namely, nitrogen fertilization rates (NFRs) and planting dates (PDs). The NFRs included the control (N_0_) with no applied N and 30 kg N ha^−1^ (N_30_), 60 kg N ha^−1^ (N_60_), and 90 kg N ha^−1^ (N_90_) urea applied. Targeted rice planting windows were last weeks of August, September, and October of each season. However, it was not always possible to perform planting on targeted dates in the second season (2019–2020) and was delayed because of unfavorable field conditions. Planting dates for 2018 were 30 August, 26 September, and 31 October 2018 for the early (PD1), intermedium (PD2), and late (PD3) planting in the first growing season (2018–2019), and 1 September, 6 October, and 3 November for the early (PD1), the intermedium (PD2) and late (PD3) planting dates in 2019 in the second growing season (2019–2020), respectively. The genotype used in experiments was Dawk Pa-yawm, which is a non-glutinous (Suwanasa et al., 2018). Thai upland rice genotype, very popular because of its aromatic fragrance, and is commonly cultivated in upland rice-growing areas of Thailand. Before planting in both seasons, the experimental field was plowed twice using a disc plow and a rotavator (twice). Experimental treatments were arranged in a randomized complete block design with three replicates. Each treatment was designated in an individual plot (3 × 3 m^2^) having 11 rows with 30-cm row-to-row spacing. To reduce the risk of lateral movement of nutrients during high rainfall intervals, all the plots were separated by a 1.5-m buffer space on each side with 0.3-m-high dikes. Drain furrows were made in the center of the surrounding buffer space of each plot, and during heavy rainfall, the dikes were cut to drain excess rainwater from each plot to avoid overflow of rainwater. In both seasons, the recommended basal fertilizer rate (DRRD, 2017) for phosphorus (19 kg P_2_O_5_ ha^−1^) and potassium (13 kg K_2_O ha^−1^) was applied equally to all the experimental plots before planting. Five seeds per hill were manually planted at 5-cm soil depth using a hand hoe, maintaining a 25-cm plant-to-plant distance. A sprinkler irrigation system with a sprinkler head (ANT-1401, product code: 351-1401160®), a water discharge capacity of 160 L h^−1^, and a 3-m diameter of water dispersal range was installed for supplementary irrigation (10–15 mm per event) at the time of planting and during the hot and dry intervals of each growing season. Thinning was performed to maintain a single plant per hill after 20–25 days of germination to attain a uniform plant stand. Urea (46% N) was used as a source of N fertilizer and was applied in two uniform splits by incorporating the fertilizer at ~5 cm soil depth in the plant rows using a hand-operated mini plow in the experimental plots according to the experimental design at the initiation of the tillering and panicle emergence stages. Weeds were manually removed from plots, and recommended cultural practices were used to control insects, pests, and diseases by spraying suitable chemical formulations to reduce yield losses in both seasons. The experimental field was surrounded on each side and covered with a net placed at a height of 2 m in both seasons to avoid crop damage by birds and rodents.

### Data Collections and Observations

Daily minimum and maximum temperature (°C), and daily rainfall (mm) data for both seasons were collected from Kho Hong Agrometeorology–Agricultural Information Center, Hat Yai, located 1.8 km from the experimental site. Agronomic data collection and plant sampling for determining N concentrations were performed at maturity during the harvest for each planting date. The number of days to flowering and days to maturity was recorded when 50% of flowering occurred, and 50% of plants reached physiological maturity in each experimental plot. Stem height was recorded from the ground surface to the topmost panicle or leaf. Stem density was counted at the time of maximum stem formation stage, and stems/tillers having at least one visible leaf were included. Plants from a 1-m^2^ area in each plot were manually harvested and used to record grain yield and yield components. Eight sample hills were manually harvested from the demarcated area to determine aboveground biomass yield. Plants from border rows were not harvested in each plot to avoid border effect. Rice straw and grain samples were dried in an oven at 65°C at various time intervals until constant weight (Yousaf et al., 2016), and dry weights of samples were obtained to determine grain yield and aboveground biomass.

### Nitrogen Concentration, Nitrogen Uptake, and Nitrogen Use Efficiency

Grain and straw samples were collected from each replication and treatment in both seasons. The straw samples were first chopped, and then the straw and grain samples were oven-dried at 65°C to a constant weight (Yousaf et al., 2016). The oven-dried grain and straw samples were ground to 1 mm using a grinder model “Retch Cyclone Mill Twister” (Hussain et al., 2021a). The Kjeldahl method (Kjeldahl, 1883) was used to determine concentrations at the Central Analytical Laboratory of Faculty of Natural Resources, Prince of Songkla University, Thailand. Grain and straw N uptake for each treatment was computed by multiplying grain yield and straw yield with corresponding N concentrations (Abbasi et al., 2012; Hammad et al., 2017). Nitrogen use efficiency (NUE) for agronomic N efficiency [AE_N_; increase in grain yield (kg) relative to applied N (kg)] of applied N (Equation 1), nitrogen recovery efficiency (RE_N_; N absorbed and used by plant) (Equation 2), partial factor productivity (PFP: ratio between grain yield and amount of fertilizer applied N uptake) (Equation 3), and nitrogen harvest index (Equation 4) were computed as outlined by Wang et al. (2018).

(1) $$\begin{array}{r} \text{A}{\text{E}}_{\text{N}}\;=\;\frac{\text{grain yield }\left({\text{N}}_{\text{x}}\right)\;-\text{ grain yield }\left({\text{N}}_{0}\right)}{\text{ N fertilizer applied }\left({\text{N}}_{\text{x}}\right)} \end{array}$$

(2) $$\begin{array}{r} \text{R}{\text{E}}_{\text{N}}\;=\;\frac{\text{N uptake }\left({\text{N}}_{\text{x}}\right)\;-\text{ N uptake }\left({\text{N}}_{0}\right)}{\text{N fertilizer applied }\left({\text{N}}_{\text{x}}\right)}\;\times \;100 \end{array}$$

(3) $$\begin{array}{r} \text{PFP }=\;\frac{\text{grain yield }}{\text{N fertilizer applied}} \end{array}$$

(4) $$\begin{array}{r} \text{NHI }=\;\frac{\text{grain N content}}{\text{total plant N uptake}} \end{array}$$

### Water Input and Crop Water Productivity

Irrigation water data for each PD were recorded with the amount of water applied using a sprinkler irrigation system. Daily rainfall data were collected from Kho Hong Agrometeorology–Agricultural Information Center, Hat Yai, Office of the Thai Meteorological Department. Irrigation water and rainfall received during each PD were added to calculate the total water input. Crop water productivity (kg m^−3^) was computed by dividing grain yield (kg) by total water input (m^3^) using Equation (5) as described by Liu et al. (2019) and Zhou et al. (2017).

(5) $$\begin{array}{r} \text{Crop water productivity }\left(\text{kg }{\text{m}}^{-3}\right)\;=\;\frac{\text{Grain yield}}{\text{Total water input}} \end{array}$$

### Economic Assessment

Economic assessment for profitability based on grain yield (kg ha^−1^) obtained from N fertilization relative to non-N fertilized plots was performed. Urea was used as a N fertilizer source, and N application cost for NFR was computed based on the prevailing market price (800 Thai Baht = US$ 23.76 per 50-kg bag) of urea fertilizer. Dawk Pa-yawm rice product selling price of $ 1.78 per kg (Thai Baht 60 = US$ 1.78 per kg.) was taken from farmer's market, and used in an economic assessment. Marginal benefit-cost ratio (MBCR), which provides the marginal assessment of economic returns of various treatments, was computed (Equation 6) (Rahman et al., 2011; Anwar et al., 2021):

(6) $$\begin{array}{r} \text{MBCR }=\;\frac{\text{Gross retur}{\text{n}}_{\text{N added}}\;-\text{ Gross retur}{\text{n}}_{\text{control}}}{\text{Gross cos}{\text{t}}_{\text{N added}}\;-\text{ Gross cost}{\;}_{\text{control}}} \end{array}$$

### Statistical Analysis

The analysis of variance (ANOVA) function of statistical package Statistix 8.1 (Tallahassee, FL, United States) (Duangpan et al., 2022) was used for statistical analysis and to evaluate the effects of applied N treatments, PDs, seasons, and their interactions. Least significant difference (LSD) was used for mean comparisons at a 5% probability level. The relationship among applied NFRs, agronomic attributes of upland rice, N uptake, and crop water productivity was evaluated by regression analysis using Statistix 8.1 and Microsoft Excel (Santiago-Arenas et al., 2021). A correlation analysis was performed to determine the association among the studied attributes, and the “Corrplot” package (Wei and Simko, 2021) of the R program (Core Team, 2021) was utilized to compute Pearson's correlation coefficients and visuals. The “ggplot2” package (Wickham, 2016) was used for the graphical output of boxplots for computed NUEs.

## Results

### Weather

**Figure 1:**
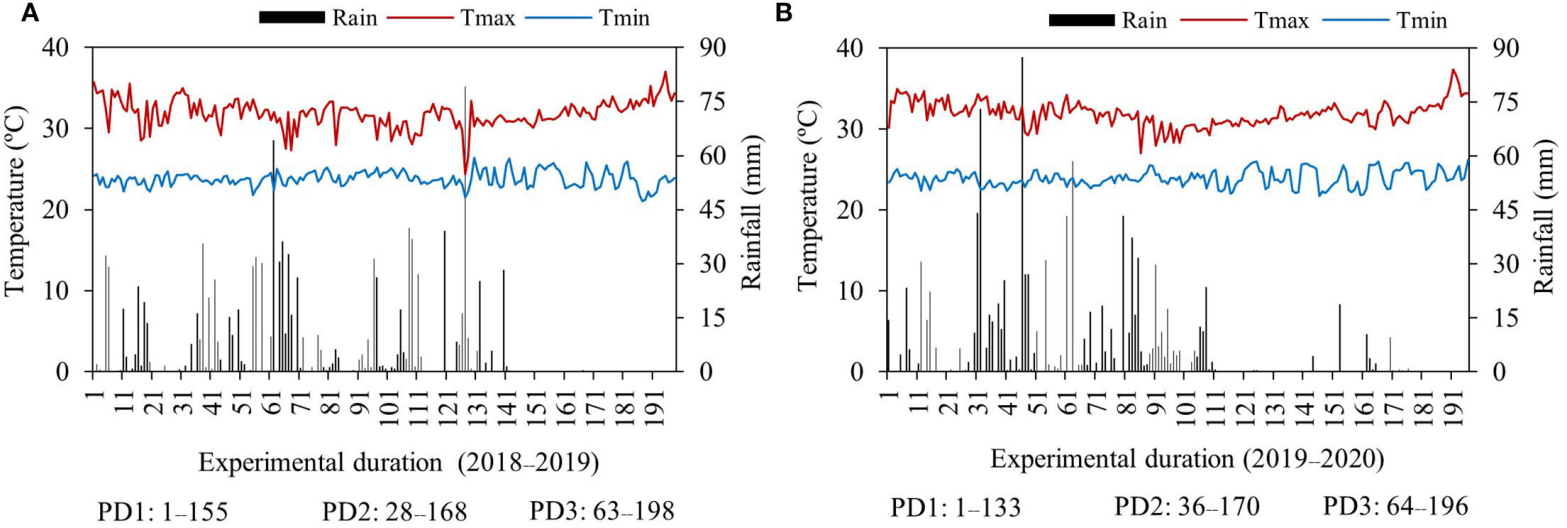
Mean daily maximum (Tmax), minimum temperature (Tmin), and daily rainfall during the experimental growing period (days) of the first season, **(A)** 2018–2019, and the second season, **(B)** 2019–2020. PD, planting date. (data source: Kho Hong; Hat Yai Agrometeorology–Agricultural Information Center: Thai Meteorological Department, Thailand).

Mean daily maximum and minimum temperatures ranged from 24 to 37 and 21 to 26°C during the first season and 27–37 and 22–26°C during the second season, respectively (Figure 1). The mean maximum and minimum temperatures were similar within respective PDs during both seasons. However, the mean maximum and minimum temperatures were slightly different from planting to flowering and from flowering to maturity during each PD in both seasons. Mean maximum temperature was higher in the first season, and minimum temperature was comparatively higher in the second season for PD3 (Table 1). The highest total rainfall received during PD1 was 1,152 mm during the first season and 1,061 mm during the second season, whereas PD2 and PD3 received 997 and 652 mm during the first season and 823 and 444 mm during the second season, respectively (Figure 1). However, the rainfall distribution from planting to flowering and from flowering to the physiological maturity period of each planting date was highly variable. Early planting (PD1) received 921- and 1,060-mm rainfall during the planting to flowering period of the first and second seasons, respectively. Intermediate planting (PD2) received 966- and 777-mm rainfall during the sowing to flowering period of the first and second seasons, respectively. Late planting (PD3) received 652- and 443-mm rainfall, comparatively less than PD1 and PD2 during the sowing to flowering period of the first and second seasons, respectively. From flowering to physiological maturity, PD1 received 231 mm as the highest rainfall during the first season. However, during the second season, PD1 received only 1-mm rain because of delayed planting, while PD2 received 31- and 46-mm rainfall from the flowering to physiological maturity period during the first and second seasons, respectively. During the flowering to physiological maturity period in the first season, delayed planting at PD3 did not receive any rainfall, and in the second season, only 1-mm rainfall occurred (Table 1). High temperatures and most dry spells occurred during the growing period of delayed planting on PD3 in both seasons.

**Table 1:** Mean maximum temperature (Tmax), minimum temperature (Tmin), and total rainfall from planting to flowering and flowering to physiological maturity of each planting date (PD) during the growing seasons of 2018–2019 and 2019–2020.

	**Planting date**	**Tmax (°C)**		**Tmin (°C)**		**Rainfall (mm)**		**Irrigation (mm)**	
**Duration**		**2018–2019**	**2019–2020**	**2018–2019**	**2019–2020**	**2018–2019**	**2019–2020**	**2018–2019**	**2019–2020**
Planting to flowering	PD1	31.8	31.8	23.8	23.8	921	1060	75	75
	PD2	31.4	31.2	23.9	23.8	966	777	55	135
	PD3	31.1	31.2	24.0	23.9	652	443	155	245
Flowering to physiological maturity	PD1	30.8	31.0	23.9	24.1	231	1	30	20
	PD2	31.4	31.9	24.1	23.4	31	46	40	20
	PD3	33.5	33.4	23.3	24.5	0	1	50	60

### Crop Performance and Effect of Season

**Figure 2:**
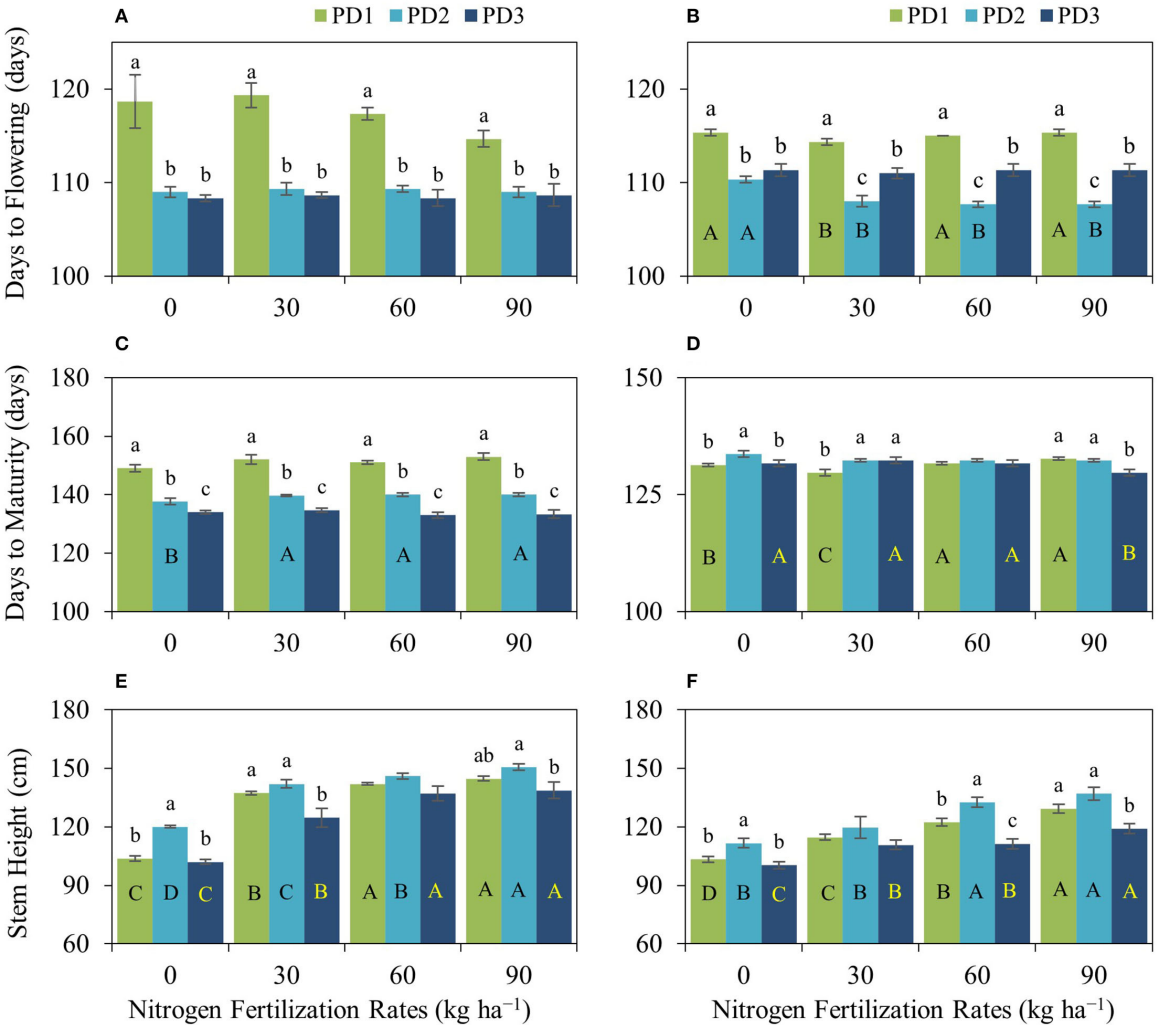
Impact of nitrogen (N) fertilization rates and planting dates on **(A,B)** days to flowering, **(C,D)** days to maturity, and **(E,F)** stem height of upland rice during the first season, **(A,C,E)** 2018–2019, and the second season, **(B,D,F)** 2019–2020. Significance (*p* < 0.05) for the means (± standard errors of 3 replicates) of traits under various N fertilization rates within each planting date is indicated by uppercase letters inside the respective bars. Significance (*p* < 0.05) for the means (± standard errors of 3 replicates) of traits under various planting dates within each N fertilization rate is indicated by lowercase letters above the bars. Bars without letters refer to a non-significant difference (*p* ≥ 0.05).

**Figure 3:**
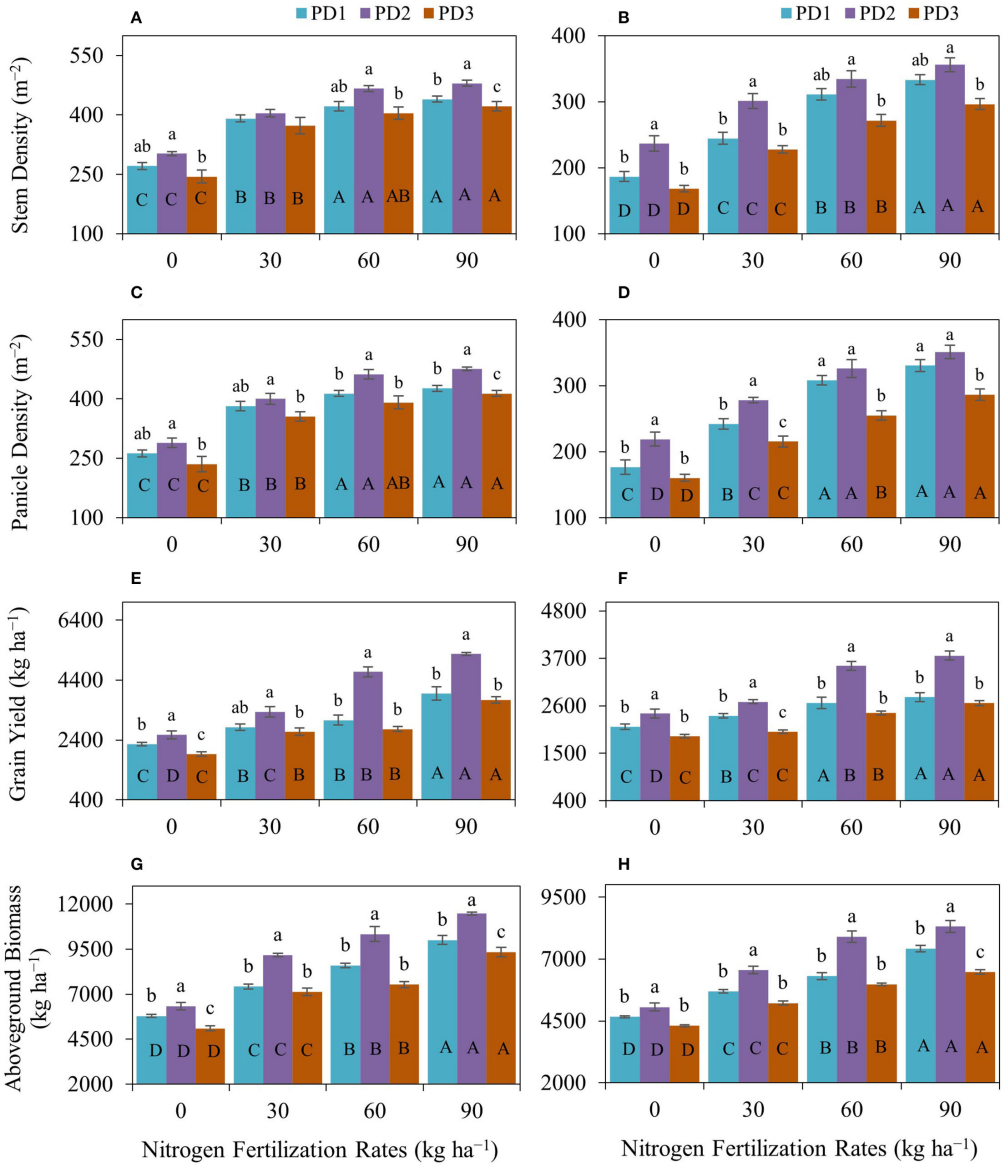
Impact of nitrogen (N) fertilization rates and planting dates on **(A,B)** stem density, **(C,D)** panicle density, **(E,F)** grain yield, and **(G,H)** aboveground biomass of upland rice (genotype: Dawk Pa–yawm) during the first season, **(A,C,E,G)** 2018–2019, and the second season, **(B,D,F,H)** 2019–2020. Significance (*p* < 0.05) for the means (± standard errors of 3 replicates) of traits under various N fertilization rates within each planting date is indicated by uppercase letters inside the respective bars. Significance (*p* < 0.05) for the means (± standard errors of 3 replicates) of traits under various planting dates within each N fertilization rate is indicated by lowercase letters above the bars.

The statistical analysis indicated that various NFRs, under the effect of seasons (S) and in the interaction of NFR × planting date (PD), NFR × S, and NFR × PD × S, did not significantly affect days to flowering. However, PD alone and in the interaction with the seasons (PD × S) significantly influenced day to flowering, whereas the interactions of NFR and PD were not significantly different. Similarly, N fertilization under various NFRs alone and in the interaction of NFR, PD, and S did not significantly affect days to maturity. However, days to maturity was significantly influenced under the effect of PD, S, NFR × PD, NFR × S, and PD × S. Stem height, stem density, and panicle density acted similarly and were significantly affected under NFR, PD, S, and in the interaction of NFR × S, whereas they were not significantly influenced under NFR × PD, PD × S, and NFR × PD × S. Grain yield and aboveground biomass acted similarly and were affected significantly under NFR, PD, S, NFR × PD, NFR × S, and; PD × S, whereas they were not influenced under the combined interaction of NFR, PD, and S (Table 2). The effect of season was significantly different in both seasons (Table 2). Rainfall occurrence and distribution were higher and comparatively suitable in the first season (Figure 1). Crop growth and maturity duration were extended (Figure 2); therefore, the upland rice performed better in the first season than in the second season (Figure 3).

**Table 2:** F–values and significance obtained from the combined analysis of variance for days to flowering, days to maturity, stem height, stem density, panicle density, grain yield, aboveground biomass, straw nitrogen (N) content, grain N content, total plant N uptake, and crop water productivity of upland rice (genotype: Dawk Pa–yawm) as influenced by various N fertilization rates (NFRs) and planting dates.

**Traits**	**Nitrogen fertilization rate (NFR)**	**Planting date (PD)**	**Season (S)**	**NFR × PD**	**NFR × S**	**PD × S**	**NFR × PD × S**
Days to flowering	1.59^ns^	173.55^***^	0.22^ns^	0.79^ns^	1.56^ns^	19.09^***^	1.80^ns^
Days to maturity	0.73^ns^	249.87^***^	901.71^***^	2.93^*^	3.71^*^	267.98^***^	1.32^ns^
Stem height	147.51^***^	58.88^***^	181.63^***^	0.90^ns^	12.27^***^	0.02^ns^	1.94^ns^
Stem density	416.23^***^	106.10^***^	1,141.89^***^	0.34^ns^	14.64^***^	1.46^ns^	1.93^ns^
Panicle density	424.2^***^	108.95^***^	1,122.01^***^	0.61^ns^	13.98^***^	1.20^ns^	1.50^ns^
Grain yield	216.73^***^	194.00^***^	212.01^***^	12.58^**^	27.77^***^	6.25^**^	1.88^ns^
Above ground biomass	602.11^***^	271.30^***^	1,046.98^***^	8.87^**^	35.13^***^	8.00^**^	1.47^ns^
Straw N content	1,211.02^***^	958.70^***^	1,927.22^***^	29.97^***^	101.56^***^	44.06^***^	3.24^**^
Grain N content	447.25^***^	909.10^***^	404.46^***^	34.16^***^	41.66^***^	38.43^***^	3.93^**^
Total plant N uptake	1,129.38^***^	1,491.77^***^	1,393.23^***^	50.28^***^	99.25^***^	64.95^***^	3.62^**^
Crop water productivity	228.78^***^	246.59^***^	53.31^***^	12.80^***^	21.61^***^	3.01^ns^	1.93^ns^

*** *p < 0.001,*

** *p < 0.01,*

* *p < 0.05,*

ns *, non-significant at p ≥ 0.05*.

#### Phenology

Phenology was not significantly influenced by N fertilization. However, flowering in PD1 during the first season crop under N_90_ occurred 4 days earlier than under N_0_; whereas maturity was delayed 2–4 days under N fertilization compared to N_0_. PD significantly affected phenology, and days to flowering and maturity were decreased by 6–11 and 15–20 days, respectively, under PD2 and PD3 at various NFRs except for days to maturity during the second season (Figures 2A–D). Crop duration was relatively shorter in the second season than in the first season possibly because of prevailing climatic conditions.

#### Stem Height

NFR and PD significantly affected stem height during both seasons. Stem height was increased with an increase in NFR, and maximum stem height was observed at N_90_ under all the PDs during both seasons. Increase in stem height under the effect of N addition ranged 33–40% under PD1, 18–26% under PD2, and 23–36% under PD3 during the first season and 11–25% under PD1, 7–23% under PD2, and 10–19% under PD3 during the second season. Under the influence of PDs, maximum stem height was observed in PD2 at all NFRs during both seasons. Stem height increased by 15, 4, 3, and 4% for N_0_, N_30_, N_60_, and N_90_, respectively, under PD2 during the first season, while it was increased by 8, 4, 8, and 6% for N_0_, N_30_, N_60_, and N_90_, respectively, under PD2 during the second season. Delayed planting (PD3) resulted in a decline in stem height by 15, 12, 6, and 8% for N_0_, N_30_, N_60_, and N_90_, respectively, under PD3 during the first season, and by 11, 8, 19, and 15% for N_0_, N_30_, N_60_, and N_90_, respectively, under PD3 during the second season (Figures 2E,F). PD alone had a significant positive impact on stem height.

#### Stem and Panicle Density

Stem density was positively influenced by N addition and N fertilization at the initiation of tillering stage, resulted in increased number of secondary stems and stem density (m^−2^) in both seasons (Figures 3A,B). Maximum stem density was observed at N_90_ under all the PDs during both seasons. The increase in stem density under the effect of N fertilization ranged from 44 to 62% under PD1, 34–59% under PD2, and 53–73% under PD3 during the first season and were 31–79% under PD1, 27–50% under PD2, and 35–76% under PD3 during the second season. Maximum stem density was observed under PD2 at all the NFRs during both seasons and increased by 11, 3, 10, and 9% in the N_0_, N_30_, N_60_, and N_90_ treatments, respectively, during the first season, and increased by 27, 23, 8, and 7 in N_0_, N_30_, N_60_, and N_90_, respectively, during the second season. Delayed planting (PD3) resulted in decline in stem density by 19, 8, 13, and 12% in N_0_, N_30_, N_60_, and N_90_, respectively, during the first season, and by 29, 25, 19, and 17% in N_0_, N_30_, N_60_, and N_90_, respectively, during the second season. Similarly, panicle density was positively influenced by N addition. Nitrogen fertilization resulted in increased panicle density (m^−2^) in both seasons (Figures 3C,D). Maximum panicle density was observed at N_90_ under all the PDs during both seasons. The increase in panicles under the effect of N fertilization ranged from 46 to 63% under PD1, 38–65% under PD2, and 51–76% under PD3 during the first season, and ranged from 37 to 87% under PD1, 27–60% under PD2, and 34–79% under PD3 during the second season. Under the influence of PDs, maximum panicle density (m^−2^) was observed under PD2 and at all the NFRs during both seasons, and it increased by 10, 5, 12, and 11% for N_0_, N_30_, N_60_, and N_90_, respectively, during the first season, whereas it was increased by 24, 15, 6, and 6% in N_0_, N_30_, N_60_, and N_90_, respectively, during the second season. Delayed planting (PD3) resulted in decline in panicle density (m^−2^) by 18, 11, 15, and 13% in N_0_, N_30_, N_60_, and N_90_, respectively, during the first season, and by 27, 23, 22, and 19% in N_0_, N_30_, N_60_, and N_90_, respectively, during the second season. PD significantly impacted stem density and panicle density (Figures 3A–D). Both attributes were increased under PD2 and decreased under PD3 with N_0_. Maximum increase under PD2 and maximum decrease under PD3 in stem density were observed with N_0_ during both seasons. Maximum increase in stem density and panicle density was observed with N_0_ in both seasons.

**Figure 4:**
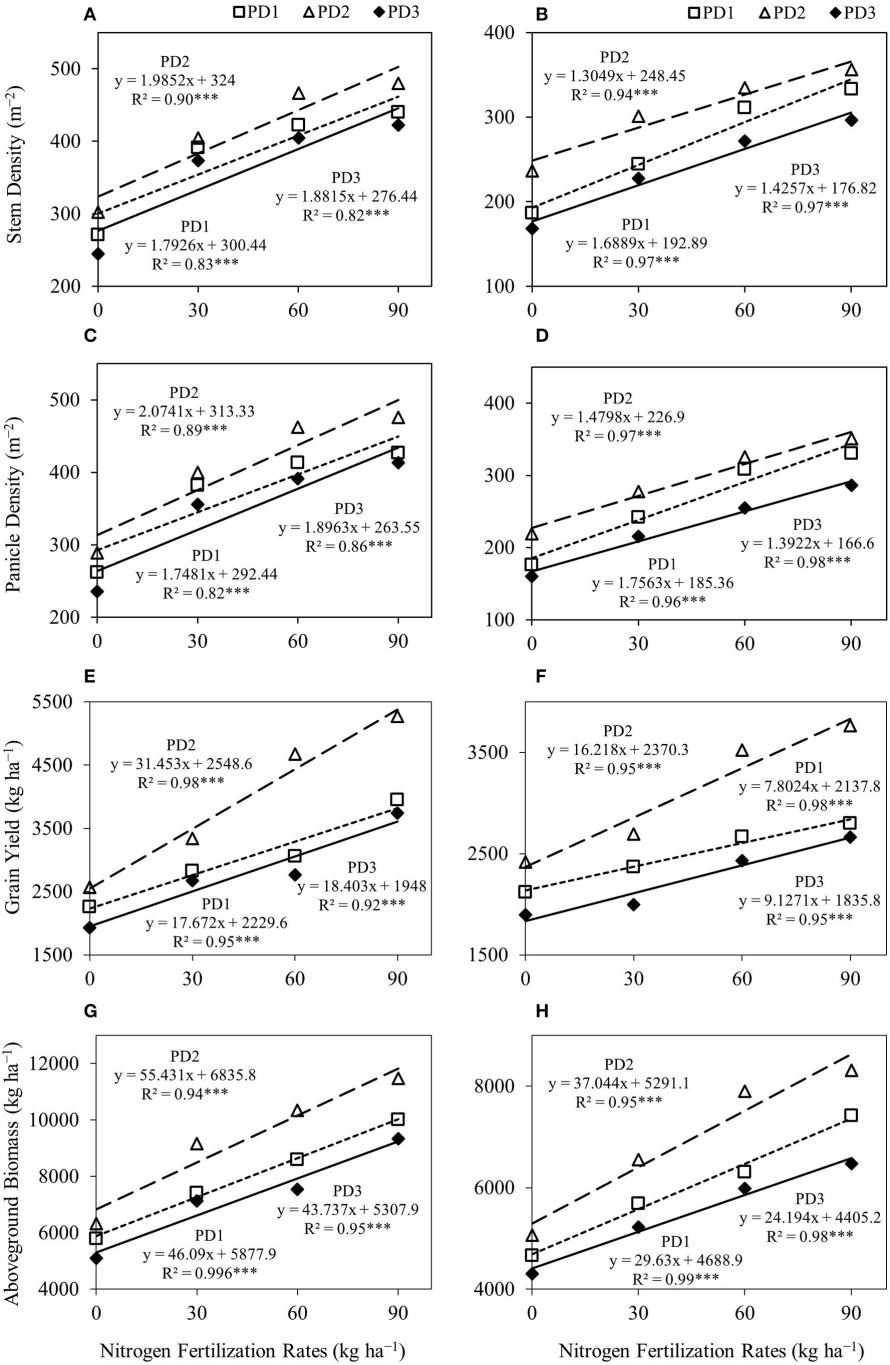
Linear regression relationships **(A,B)** between nitrogen (N) fertilization rates and stem density, **(C,D)** between N fertilization rates and panicle density, **(E,F)** between N fertilization rates and grain yield, and **(G,H)** between N fertilization rates and aboveground biomass for upland rice obtained from the first season, **(A,C,E,G)** 2018–2019, and the second season, **(B,D,F,H)** 2019–2020, data. *** Significant at *p* < 0.001.

The regression analysis for stem density (Figures 4A,B) and panicle density (Figures 4C,D), and NFR under all the PDs indicated a highly significant linear relationship between both seasons and stem density, as well as panicle density, continued to increase with increase in NFR.

#### Grain Yield and Aboveground Biomass

Grain yield was positively affected by varying N additions on all the planting dates, and maximum grain yield was obtained with N_90_ in both seasons (Figures 3E,F). Grain yield was increased by 25–75% at PD1, 30–105% at PD2, and 38–94% at PD3 during the first season and by 12–32% at PD1, 11–56% at PD2, and 5–41% at PD3 during the second season. Maximum grain yield was obtained in PD2 at all the NFRs in both seasons, and it was increased by 14, 18, 53, and 33% in N_0_, N_30_, N_60_, and N_90_, respectively, under PD2 during the first season, whereas it was increased by 14, 14, 32, and 34% for N_0_, N_30_, N_60_, and N_90_, respectively, under PD2 during the second season. A decline in grain yield was observed because of delayed planting at PD3 as compared to PD2, and grain yield was decreased by 25, 20, 41, and 29% for N_0_, N_30_, N_60_, and N_90_, respectively, at PD3 during the first season, whereas it was decreased by 22, 26, 31, and 29% for N_0_, N_30_, N_60_, and N_90_, respectively, at PD3 during the second season. Similarly, aboveground biomass was also positively influenced by N addition, and N fertilization resulted in increased aboveground biomass in both seasons (Figures 3G,H). Maximum aboveground biomass was observed at N_90_ at all the PDs during both seasons. Nitrogen addition increased aboveground biomass by 28–73% at PD1, 45–81% at PD2, and 40–83% at PD3 during the first season, and by 22–59% at PD1, 29–64% at PD2, and 21–50% at PD3 during the second season. A decline in aboveground biomass was also observed because of delayed planting at PD3 as compared to PD2, and it was decreased by 19, 22, 27, and 19% in N_0_, N_30_, N_60_, and N_90_, respectively, at PD3 during the first season, whereas it was decreased by 15, 20, 24, and 22% for N_0_, N_30_, N_60_, and N_90_, respectively, at PD3 during the second season.

The effect of PD was considerable on grain and aboveground biomass yields under all the NFRs. Grain yield and aboveground biomass were increased at PD2 and decreased under delayed planting, PD3. The regression analysis for grain yield (Figures 4E,F) and aboveground biomass (Figures 4G,H), and NFRs under all the PDs indicated a highly significant linear relationship between in both seasons and grain yield, as well as aboveground biomass, continued to increase with increase in NFR in this assessment.

### Nitrogen Uptake

**Figure 5:**
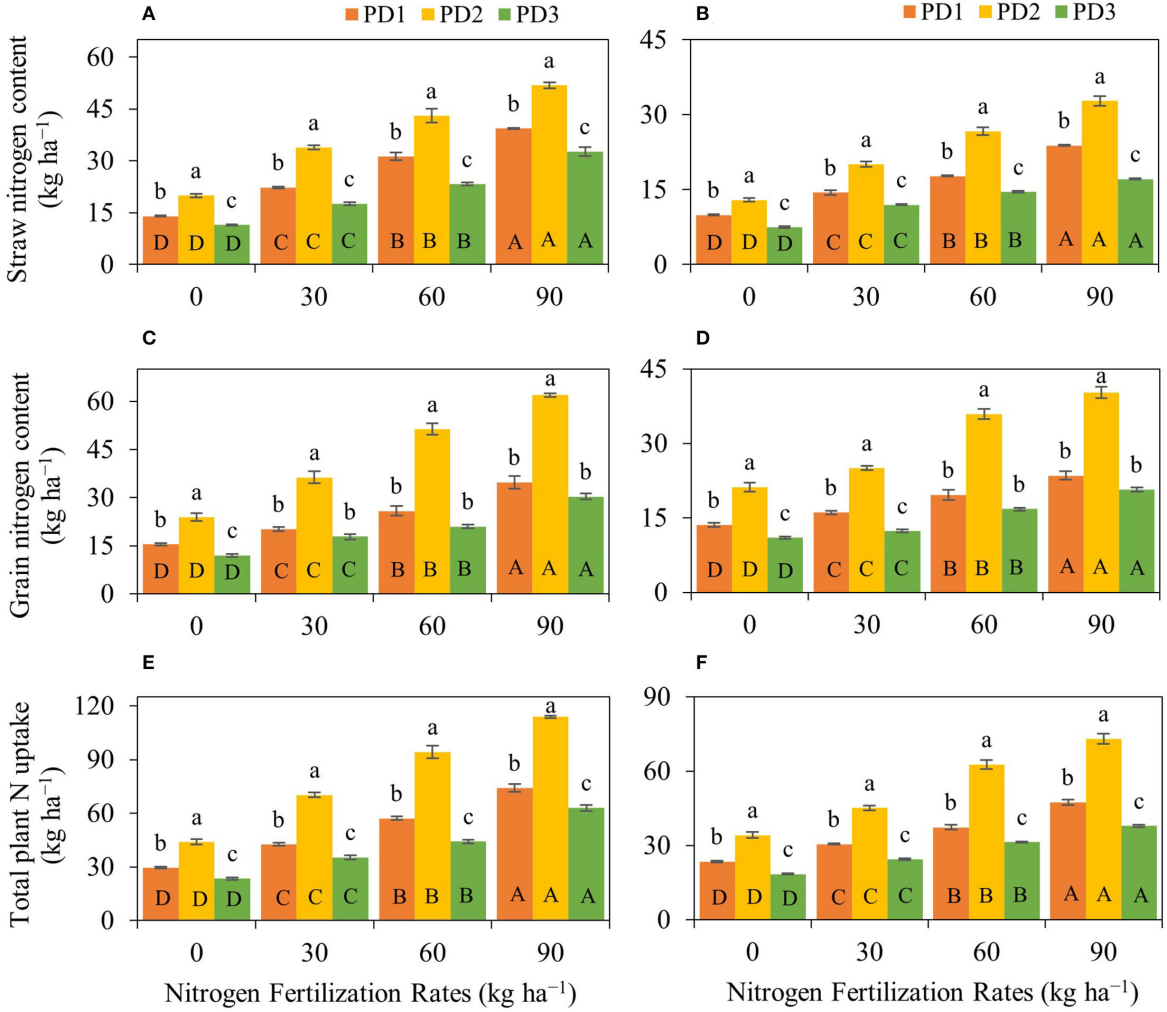
Impact of nitrogen (N) fertilization rates and planting dates on **(A,B)** rice straw N content, **(C,D)** rice grain N content, and **(E,F)** total plant N uptake of upland rice during the first season, **(A,C,E)** 2018–2019, and the second season, **(B,D,F)** 2019–2020. Significance (*p* < 0.05) of the means (± standard errors of 3 replicates) with respective attribute under various N fertilization rates within each planting date is indicated by uppercase letters inside the respective bars. Significance (*p* < 0.05) of the means (± standard errors of 3 replicates) with respective attribute under various planting dates within each N fertilization rate is indicated by lowercase letters above the bars.

**Figure 6:**
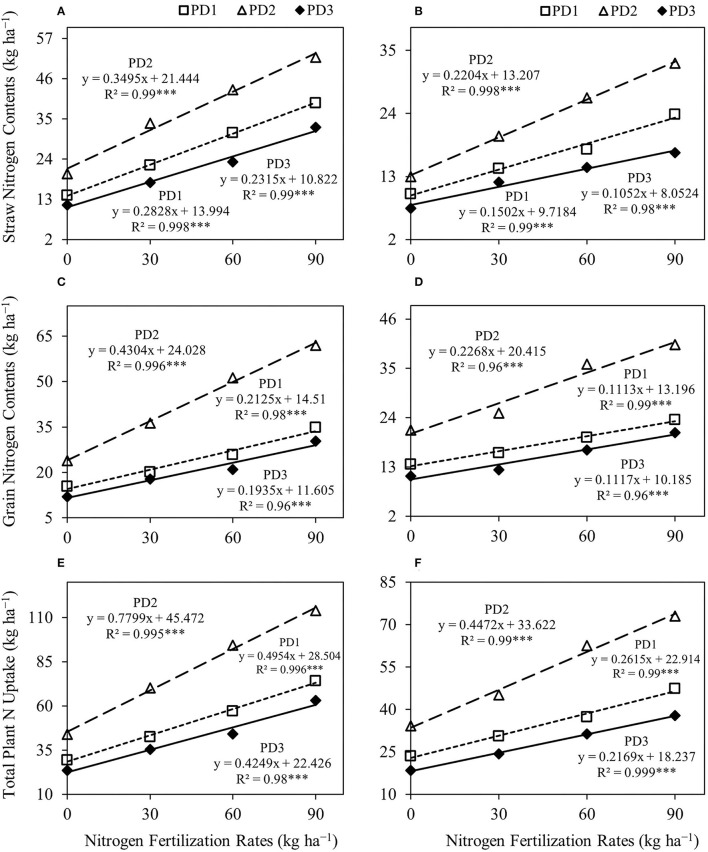
Linear regression relationships **(A,B)** between nitrogen (N) fertilization rates and straw N contents, **(C,D)** between N fertilization rates and grain N contents, and **(E,F)** between N fertilization rates and total plant N uptake for upland rice obtained from the first season, **(A,C,E)** 2018–2019, and the second season, **(B,D,F)** 2019–2020, data. *** Significant at *p* < 0.001.

Rice straw N content, grain N content, and total plant N uptake significantly (*p* < 0.001) differed under the effect of treatments and their interactions, including NFR, PD, S, NFR × PD, NFR × S, PD × S, and NFR × PD × S (Table 2). A significant increase in straw, grain and total N uptake was observed with increase in NFR under all the PDs during both seasons (Figure 5). The effect of N fertilization on rice straw N content ranged from 59 to 181% at PD1, 69 to 160% at PD2 and 54 to 186% at PD3, and 45 to 140% at PD1, 55 to 153% at PD2, and 60 to 129% at PD3 during the first and the second seasons, respectively (Figures 5A,B). Under the influence of PDs, maximum straw N content was observed at PD2 and all the NFRs during both seasons. Straw N uptake increased at PD2 by 43, 52, 38, and 32%, whereas it increased by 30, 39, 51, and 37% for N_0_, N_30_, N_60_, and N_90_ during the first and second seasons, respectively, when compared to PD1. Delayed planting (PD3) resulted in decline in straw N content by 43, 48, 46, and 37% and by 42, 40, 46, and 48% for N_0_, N_30_, N_60_, and N_90_ at PD3 as compared to PD2 during the first and second seasons, respectively. A significant linear relationship was indicated between NFR and straw N content for all the planting dates in both seasons (Figures 6A,B).

Rice grain N content increased by 31–125% at PD1, 52–159% at PD2, and 48–152% at PD3 during the first season, and by 19–73% at PD1, 18–90% at PD2, and 13–88% at PD3 during the second season (Figures 5C,D). Under the influence of PDs, maximum grain N content was observed at PD2 at all the NFRs during both seasons. Grain N uptake increased by 55, 80, 98, and 78% during the first season, whereas it was increased by 57, 55, 83, and 71% for N_0_, N_30_, N_60_, and N_90_, respectively, at PD2 during the second season when compared to PD1. Delayed planting (PD3) resulted in decline in rice grain N content by 50, 51, 59, and 51% during the first season, and by 48, 51, 53, and 49% during the second season for N_0_, N_30_, N_60_, and N_90_, respectively, at PD3 when compared to PD2. Grain N content continued to increase with increase in NFR under all the PDs, and a linear relationship was observed (Figures 6C,D).

A similar trend was observed for total plant N uptake (Figures 5E,F). Total plant N uptake was increased by 44–152, 60–159, and 51–168% during the first season and by 30–102, 32–114, and 32–105% during the second season at PD1, PD2, and PD3, respectively. Under the influence of PDs, maximum total plant N uptake was observed in PD2 at all the NFRs during both seasons. Total plant N uptake was increased by 49, 65, 65, and 54% during the first season, whereas it was increased by 45, 48, 68, and 54% during the second season in N_0_, N_30_, N_60_, and N_90_, respectively, when PD2 compared to PD1. Delayed planting (PD3) resulted in decline in total plant N uptake by 20, 35, 50, and 51% during the first season, and by 46, 46, 50, and 48% during the second season in N_0_, N_30_, N_60_, and N_90_, respectively, at PD3 as compared to PD2. The regression analysis for total plant N uptake and NFR under all the PDs also indicated a highly significant linear relationship during both seasons (Figures 6E,F).

### Nitrogen Use Efficiencies

**Figure 7:**
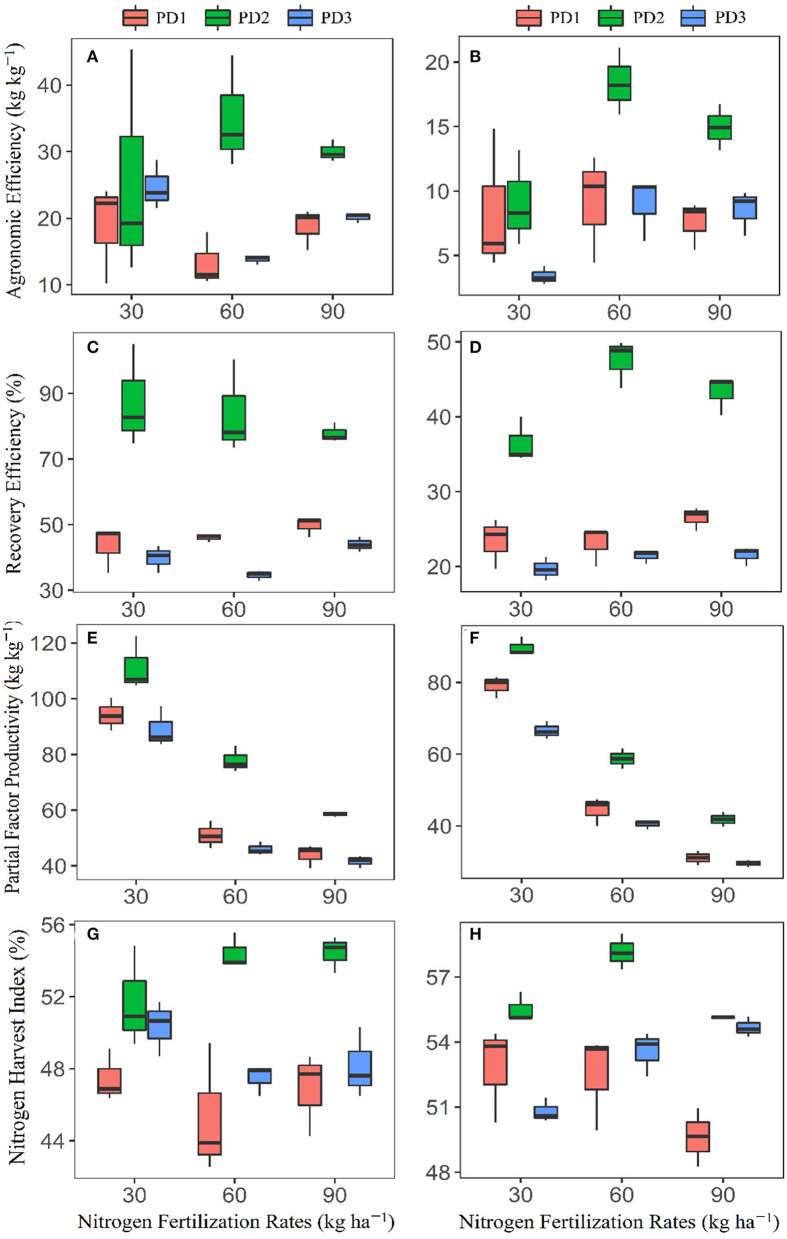
Box plots for **(A,B)** agronomic efficiency, **(C,D)** recovery efficiency, **(E,F)** partial factor productivity, and **(G,H)** nitrogen harvest index of applied nitrogen fertilizer for upland rice (genotype: Dawk Pa–yawm), as influenced under different planting dates during the first season, **(A,C,E,G)** 2018–2019, and the second season, **(B,D,F,H)** 2019–2020. Means are presented with black horizontal lines within the box plots for each planting date, whereas the upper and lower boundaries of box plots indicate 75th and 25th percentiles of data, respectively. The upper and lower whiskers indicate maximum and minimum data points.

Nitrogen fertilization influenced N use efficiencies, including agronomic efficiency (AE_N_), recovery efficiency (RE_N_), partial factor productivity (PFP), and N harvest index (NHI), and all varied under varying NFRs. Maximum AE_N_ (kg kg^−1^) was as follows: 18.8 in N_0_ and N_90_ at PD1, 35 in N_60_, at PD2, and 24.7 in N_0_ at PD3 during the first season, and 9.1 in N_60_ at PD1, 18.4 in N_60_ at PD2, and 9 at N_60_ at PD3 during the second season (Figures 7A,B). Significant variability in AE_N_ was observed under the influence of PD, and maximum AE_N_ was observed at PD2 at all the NFRs. Agronomic efficiency was increased by 36, 162, and 60% for N_30_, N_60_, and N_90_, respectively, during the first season, and it was increased by %, 102 and 97 for N_30_, N_60_, and N_90_, respectively, during the second season. Delayed planting (PD3) resulted in decline in AE_N_ by 4, 60, and 30% during the first season, and by 63, 51, and 43% during the second season in N_30_, N_60_, and N_90_ at PD3 as compared to PD2, respectively.

Recovery efficiency varied under N fertilization, and maximum RE_N_ of 50% in N_90_, 88% in N_30_, and 44% in N_90_ during the first season, and 27% in N_60_, 48% in N_60_, and 22% in N_60_ during the second season was observed, respectively, at PD1, PD2, and PD3 (Figures 7C,D). Significant variation in RE_N_ was observed under the influence of PD, and maximum RE_N_ was observed at PD2 at all the NFRs. RE_N_ was increased by 101, 82, and 57% during the first season, and by 56, 106, and 63% during the second season in the N_30_, N_60_, and N_90_, respectively. Delayed planting (PD3) resulted in decline in RE_N_ by 55, 59, and 43% during the first season, and by 46, 55, and 50% in N_30_, N_60_ and N_90_, respectively, under PD3 as compared to PT2 during the second season.

Partial factor productivity (PFP) gradually decreased with increase in NFR, and the maximum observed during the first season were 94.2, 111.4, and 89.1 kg kg^−1^, and 79 kg kg^−1^ at PD1, 89.8 kg kg^−1^ at PD2, and 66.6 kg kg^−1^ at PD3 during the second season (Figures 7E,F). PFP varied among the planting dates, and maximum PFP was also observed under PD2 at all the NFRs. PFP increased by 18, 53, and 33% during the first season, and by 14, 32, and 34% during the second season in N_30_, N_60_ and N_90_, respectively. PFP declined by 20, 41 and 29% during the first season and by 26, 31, and 29% during the second season in N_30_, N_60_, and N_90_, respectively, at PD3 as compared to PD2.

N harvest index (NHI) differed under N fertilization, and maximum NHI observed were 47% in N_30_ and N_90_ at PD1, 54% in N_60_ and N_90_ at PD2, and 50% in N_30_ at PD3 during the first season, and 53% in N_30_ and N_60_ at PD1, 57% in N_60_ at PD2, and 55% in N_90_ at PD3 during the second season (Figures 7G,H). NHI varied among the PDs, and maximum NHI was also observed at PD2 and all the NFRs. Nitrogen harvest index increased by 9, 20, and 16% during the first season and increased by 5, 9, and 11% during the second season, respectively, with the N_30_, N_60_, and N_90_ treatments. Nitrogen harvest index also declined by 3, 13, and 12% during the first season, and by 8, 7, and 1% during the second season with N_30_, N_60_, and N_90_, respectively, under PD3 as compared to PD2.

### Crop Water Productivity

**Figure 8:**
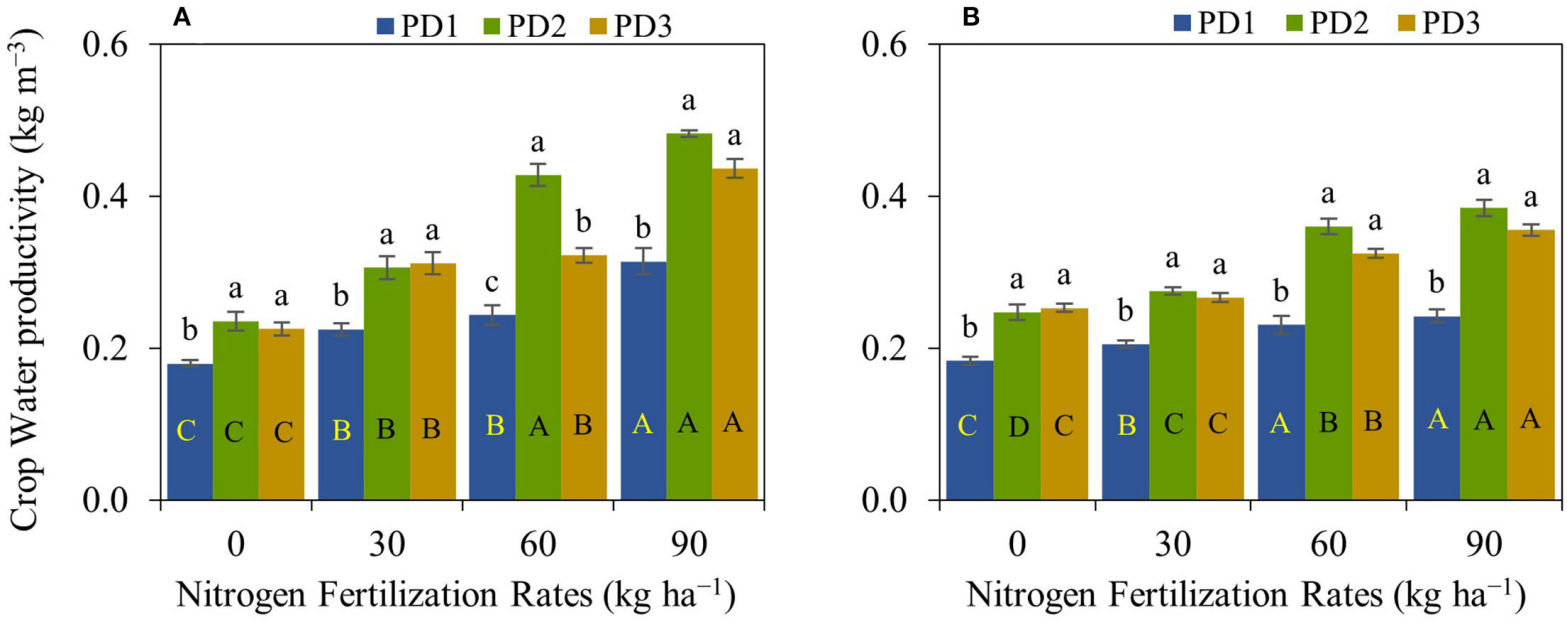
Crop water productivity of upland rice (genotype: Dawk Pa–yawm), as affected by nitrogen (N) fertilization rates and planting dates during the first season, **(A)** 2018–2019, and the second season, **(B)** 2019–2020. Significance (*p* < 0.05) for the means (± standard errors of 3 replicates) of crop water productivity under various N fertilization rates within each planting date is indicated by uppercase letters inside the respective bars. Significance (*p* < 0.05) for the means (± standard errors of 3 replicates) of crop water productivity under varying planting dates within each N fertilization rate is indicated by lowercase letters above the bars.

**Figure 9:**
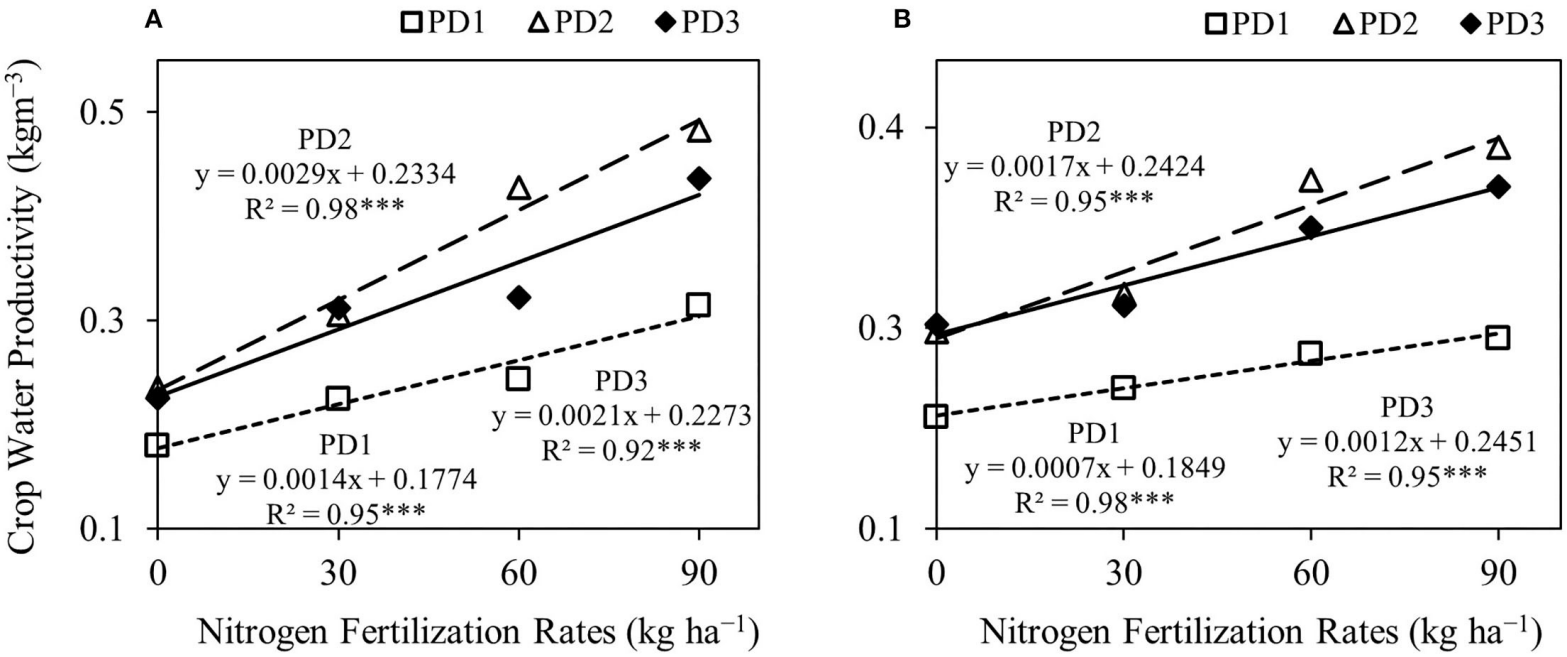
Linear regression relationships between nitrogen fertilization rates and crop water productivity **(A,B)**, of upland rice obtained from the first season, **(A)** 2018–2019, and second the season, **(B)** 2019–2020 data. *** Significant at *p* < 0.001.

Crop water productivity was significantly (*p* < 0.001) different under the effect of NFR, PD, S, NFR × PD, and NFR × S, whereas no significant interaction was observed for PD × S and NFR × PD × S (Table 2). Crop water productivity was highly influenced by seasons. An increase in crop water productivity was observed with increase in NFR, and maximum crop water productivity was at N_90_ under all the PDs during both seasons (Figure 8). The increase in crop water productivity ranged from 25 to 75, 30 to 105, and 38 to 94% during the first season, and 12–32, 11–56, and 5–41% during the second season at PD1, PD2, and PD3, respectively, under the effect of N addition. Maximum crop water productivity was observed at PD2 in all the NFRs during both seasons. Crop water productivity increased by 33, 36, 74, and 54% during the first season, and by 33, 34, 56, and 58% during the second season in N_0_, N_30_, N_60_, and N_90_, respectively, at PD2. Delayed planting (PD3) resulted in decline in crop water productivity by 4, 3, 26, and 10% during the first season, and by 4, 4, 11, and 8% during the second season in N_0_, N_30_, N_60_, and N_90_, respectively, under PD3 as compared to PD2. The regression analysis between NFR and crop water productivity under all the PDs showed a highly significant linear relationship during both seasons (Figures 9A,B).

### Pearson's Correlation

**Figure 10:**
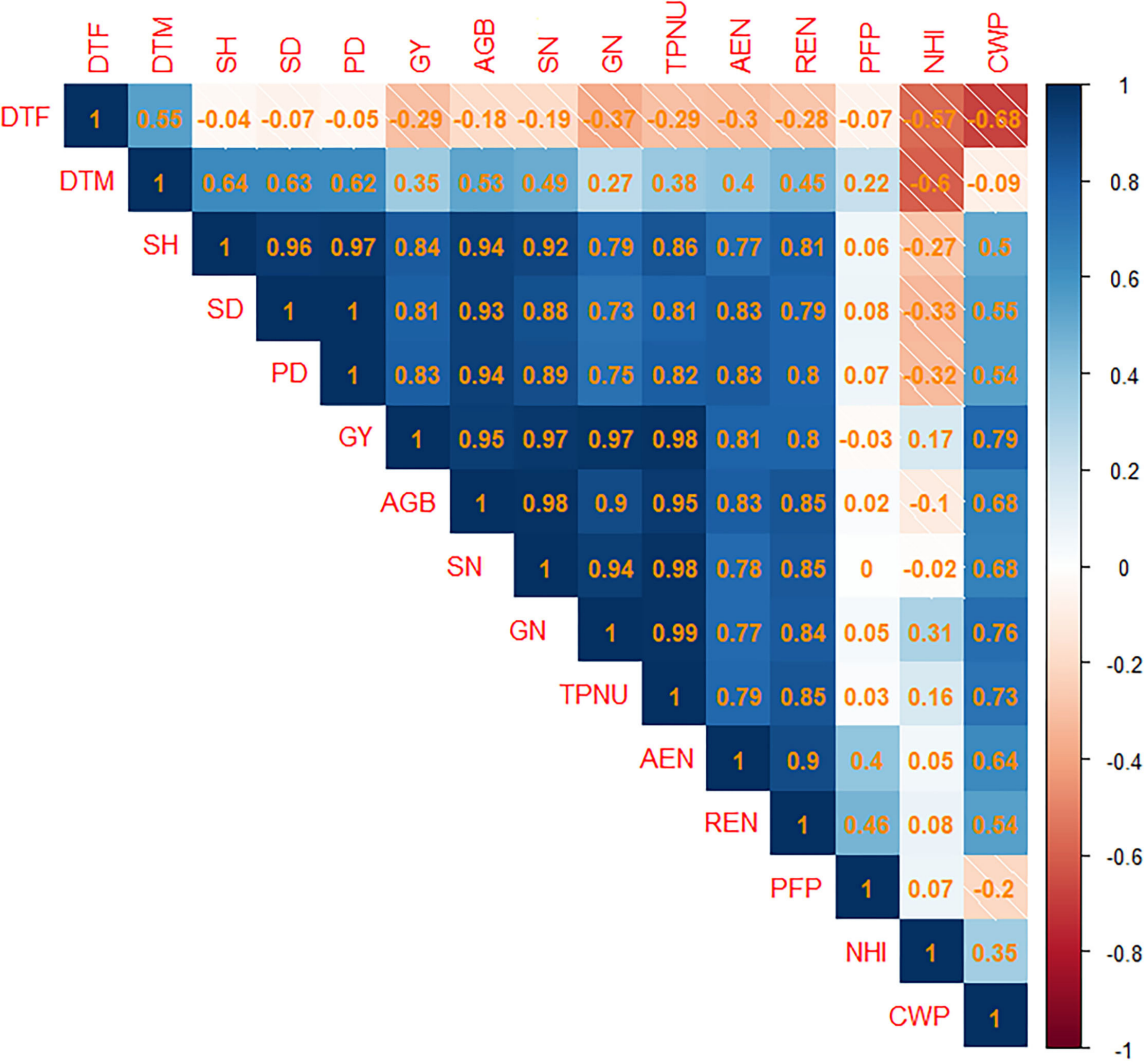
Correlation plot of Pearson's correlation analysis among the studied traits of upland rice (genotype: Dawk Pa–yawm). Blue and orange shaded squares indicate positive or negative associations among attributes. Computed Pearson's correlation coefficients are reported in the squares. The intensity of color shades is proportional to the computed coefficients. DTF, Days to flowering; DTM, days to maturity; SH, stem height; SD, stem density; PD, panicle density; GY, grain yield; AGB, aboveground biomass; SN, straw nitrogen content; GN, grain nitrogen content; TPNU, total plant nitrogen uptake; AEN, agronomic efficiency of applied nitrogen; REN, recovery efficiency of applied nitrogen; PFP, partial factor productivity; NHI, nitrogen harvest index; CWP, crop water productivity.

Pearson's correlation assessment of pooled data for studied agronomic traits of upland rice indicates a strong correlation among agronomic traits, N uptake, NUE, and crop water productivity (Figure 10). N uptake, NUE, and crop water productivity indicated the highest significant positive association between grain N content (GN) and total plant N uptake (TPNU) of 0.99. The correlation between TPNU and straw N content (SN), grain yield (GY) and TPNU, and aboveground biomass (AGB) and SN was 0.98. Correlation between stem height (SH), and panicle density = GY, and SN = GY, and GN (0.97) > SH and stem density (0.96) > GY. Furthermore, correlation of AGB = TPNU (0.95) > and AGB = SH (0.94) > SD and AGB (0.93) > AGB and GN. In contrast, the highest negative association was observed between days to flowering and crop water productivity (−0.68), and days to maturity and N harvest index (−0.6).

### Economic Assessment and Profitability

The economic analysis for Dawk Pa-yawm grain productivity per hectare indicated that increase in NFR of up to 90 kg N ha^−1^ provided the highest economic benefit for all the PDs during both seasons (Table 3). Maximum additional profits for PD1, PD2, and PD3 were 3,005.95, 4,809.15, 3,222.42 US$ ha^−1^ during the first season and 1,213.03, 2,393.62, 1,366.33 US$ ha^−1^ during the second season (Table 3). Considering the impact of planting date, profitability from applied N as compared to control was influenced by the highest gross return, and gross profit margins were observed at PD2 with N_90_ during both seasons. The highest additional profit obtained were 1,373.63, 3,741.95, and 4,809.15 US$ ha^−1^ during the first season, and 486.78, 1,966.28, and 2,393.62 US$ ha^−1^ during the second season in N_30_, N_60_, and N_90_, respectively, compared to N_0_, at PD2 (Table 3). If the Marginal benefit-cost ratio (MBCR) is considered, maximum MBCR was also observed at PD2 and it was valued at 44.32, 60.37, and 51.73 for the first season, and 15.71, 31.72, and 25.75 for the second season in N_30_, N_60_ and N_90_, respectively. An increase in grain yield productivity and profitability with an increase in nitrogen rate up to N_60_ at PD2 indicated the highest MBCR with values of 60.37 and 31.72 for the first and second season, respectively.

**Table 3:** Grain yield production, nitrogen fertilization cost, and economic return of upland rice (genotype: Dawk Pa–yawm) calculated for various nitrogen fertilization rates as affected by planting dates.

**Growing season**	**Nitrogen fertilization rate**	**Planting dates**	**Grain yield**	**Gross return**	**Nitrogen fertilization cost**	**Additional profit over control**	**Gross margin over control**	**MBCR^a^**
	**kg ha^**−1**^**		**t ha^**−1**^**	**US$ ha^**−1**^**	**US$ ha^**−1**^**	**US$ ha^**−1**^**	**US$ ha^**−1**^**	
2018–2019	0	PD1	2.26	4,024.64	–	–	–	–
		PD2	2.57	4,574.72	–	–	–	–
		PD3	1.93	3,436.05	–	–	–	–
	30	PD1	2.83	5,031.19	30.99	1,006.55	975.56	32.48
		PD2	3.34	5,948.35	30.99	1,373.63	1,342.64	44.32
		PD3	2.67	4,755.62	30.99	1,319.57	1,288.58	42.58
	60	PD1	3.06	5,450.21	61.98	1,425.58	1,363.60	23.00
		PD2	4.67	8,316.67	61.98	3,741.95	3,679.96	60.37
		PD3	2.76	4,915.69	61.98	1,479.64	1,417.66	23.87
	90	PD1	3.95	7,030.59	92.97	3,005.95	2,912.98	32.33
		PD2	5.27	9,383.87	92.97	4,809.15	4,716.18	51.73
		PD3	3.74	6,658.50	92.97	3,222.42	3,129.48	34.66
2019–2020	0	PD1	2.12	3,770.95	–	–	–	–
		PD2	2.42	4,306.48	–	–	–	–
		PD3	1.89	3,372.24	–	–	–	–
	30	PD1	2.37	4,219.25	30.99	448.30	417.30	14.47
		PD2	2.69	4,793.26	30.99	486.78	455.79	15.71
		PD3	2.00	3,554.68	30.99	182.44	151.45	5.89
	60	PD1	2.67	4,746.65	61.98	975.70	913.72	15.74
		PD2	3.52	6,272.76	61.98	1,966.28	1,904.30	31.72
		PD3	2.43	4,329.57	61.98	957.33	895.34	15.45
	90	PD1	2.80	4,983.99	92.97	1,213.03	1,120.06	13.05
		PD2	3.76	6,700.11	92.97	2,393.62	2,300.65	25.75
		PD3	2.66	4,738.57	92.97	1,366.33	1,273.36	14.70

a *MBCR, marginal benefit-cost ratio*.

## Discussion

Identification of suitable N fertilizer rate and agronomic management of N fertilizer application synchronized with ideal planting date is critical for enhancing rainfed upland rice productivity. Inadequate N fertilization during improper planting date the rice crop growth period leads to reduced N utilization efficiency and ultimately affects the productivity and profitability of upland rice production. Traditional practices of N fertilization and planting date adopted by small land-holders growing upland rice needs to be adjusted according to the soil nutrient status, upland rice N fertilizer demand, and favorable climatic conditions. Water availability during the rainfed upland rice growth period is a crucial element that can significantly influence the utilization efficiency of fertilization. Therefore, agronomic management of suitable N fertilization rate synchronized with ideal planting date is essential to enhance resource use efficiency, productivity, and profitability.

In the experimental location, the average maximum and minimum temperatures during the experimental growth period ranged from 24 to 37°C and 21 to 26°C for the first season and 27–37 and 22–26°C for the second season, respectively. According to Acquaah (2007) and Buddhaboon et al. (2011), the optimal temperature for rice growth is 27°C. The average temperature that prevailed during the rice growth period in both seasons was higher than the optimal temperature range of 25–30°C (Sparks, 2009). The average maximum and minimum temperatures were similar within respective PDs during both seasons. However, the mean maximum and minimum temperatures were different from planting to flowering and from flowering to maturity during each PD in both seasons. The mean maximum temperature was decreased with delay in planting date, whereas the mean minimum temperature was increased from the planting to flowering period. In contrast, the mean maximum temperature was increased with delay in planting date, while the mean minimum temperature was not significantly different from flowering to physiological maturity. The highest mean maximum temperature from flowering to physiological maturity was observed at PD3 during both seasons, which indicated that most hot intervals prevailed during PD3. Temperature difference significantly impacts crop growth duration, and PD regulates the use of environmental resources influencing crop performance (Varinruk, 2017). High and low temperatures occurring under changing climate affect plant growth and development (Aslam et al., 2022). Grain and biomass productivity was highly correlated to life cycle (Aslam et al., 2017). In general, crop growth duration decreased with increase in temperature because of a higher crop growth rate (Yoshida, 1973; Ahmed et al., 2014). We observed that days to flowering and days to maturity were decreased significantly in both seasons, as the temperature from flowering to physiological maturity increased under PD2 and PD3.

Rainfall distribution was different and highly variable among PDs and seasons. Maximum rainfall and high rainfall intervals occurred during PD1 in both seasons. All the PDs received maximum rainfall from planting to flowering. PD2 received a suitable distribution of rainfall during the growth period as compared to PD1 and PD3; therefore, supplementary irrigation was reduced at PD2. Due to less rainfall from planting to flowering and from flowering to physiological maturity, at PD3, supplementary irrigation was increased. As PD1 received the highest rainfall from planting to flowering and particularly from flowering to maturity, crop duration was increased because of extended plant growth and developmental phases in the first season. Previous research has confirmed that rice crop growth duration can be delayed on rainy days or during the occurrence of low temperatures in the terminal stages, and that sunny or hot days may shorten crop growth duration (GRiSP, 2013). In the second season, PD1 received maximum rainfall during the planting to flowering period and received only 1 mm from flowering to physiological maturity accompanied by higher average temperatures; hence, crop growth duration was significantly decreased.

The difference in temperature and rainfall distribution influenced crop duration and supplementary irrigation. We observed that PD1 received the highest rainfall; thus, maximum runoff and flash events occurred during PD1, whereas PD2 received a moderate distribution of rain, which was favorable as compared to PD1 and PD3. Therefore, to enhance the utilization of rainwater, slight delays in planting would be advantageous, which not only prevent heavy runoff events but also causes better rainwater distribution for plants. Luo et al. (2022) reported similar results in crop water requirement and irrigation demand for rice. The early rice required less irrigation frequency and may not require additional irrigation, while middle and late rice planting required increased water demand. In addition, an assessment of climate change impact predicted that a 30-day delay in planting of the Thai rice KDML−105 cultivar would enhance yield by 23% in the 2050s (Babel et al., 2011). The results from our study and findings of Luo et al. (2022) and Babel et al. (2011) strongly support that adjustment in PD would help to enhance natural resource use efficiency, particularly the optimal use of rainfall, with the benefit of reduced or even no supplementary irrigation.

In this study, the agronomic performance of upland rice was positively influenced by N fertilization. According to Zhang et al. (2020), N addition positively impacts plants' photosynthesis and physiological mechanisms, which determines yield. We observed that upland rice was responsive to NFR and N fertilization at various NFRs, resulted in increased stem height under all the PDs in both seasons. The synergy between NFR and stem height was well reported (Millard, 1988; Wu et al., 2020) because of the effective role of N fertilization in cell growth and enhancement of stem enlargement. Zhang et al. (2020) also found that increase in N fertilization positively influenced the stem height of rice plants. Similar findings were also reported by Jahan et al. (2020). They stated that the stem height of rice plants was enhanced by increased N fertilization. Stem height, under the influence of PD, was negatively affected, and lowest stem height was observed at PD3 at all the NFRs during both seasons. This was possibly due to higher temperatures and low rainfall during PD3, as most of the hot and dry intervals occurred during PD3. Rice is highly vulnerable and sensitive to water stress (Singh et al., 2017), and the decline in stem height of rice is well-documented under water stress (Ichsan et al., 2020; Hussain et al., 2021a,b). A positive correlation prevails among NFR, stem density, and panicle density of rice. We observed that N fertilization positively influenced stem density and panicle density under all the PDs in both seasons. According to Chen et al. (2020), high N input resulted in increased stem buds' growth, which increased stem density. Stem density and increased tillering contribute and determine panicle density. Nitrogen fertilization increased panicle density, and results were supported by the findings of Jahan et al. (2020), who confirmed in their study that higher N availability triggered cell division and caused increase in panicle density (m^−2^). In contrast, stem density and panicle density were decreased at PD3 under the influence of PD. A decline in stem density, as well as panicle density, possibly occurred because of the overall less rainfall (planting to maturity) and high temperature (particularly from flowering to maturity) that prevailed during PD3. Prevalence of slightly higher temperature above the ambient temperature results in triggered growth of rice, higher stem height, and stem density (Yoshida, 1973; Dubey et al., 2018). However, if higher temperature prevails during active stem formation stages, it results in decline in panicle density (Dubey et al., 2018). In addition, limited rainfall occurrence during PD3 possibly resulted in low soil water status, which might have induced mild water stress. The decline in stem density (Zain et al., 2014) and particularly panicle density (Davatgar et al., 2009) of rice under water stress is also well explored (Hussain et al., 2021b). Increase in stem height and stem density contributes to aboveground biomass (Hussain et al., 2021a), while increase in panicle density contributes to grain yield (Dubey et al., 2018). Nitrogen addition increased the performance of yield attributes, consequently increased grain yield and aboveground biomass. An increase in grain yield with increased NFR was also reported by Zhang et al. (2020), while Chen et al. (2020), as well as Jahan et al. (2020), also reported similar results for increase in grain yield as well as aboveground biomass under increased N supply.

Pearson's correlation assessment also revealed significant, strong, and positive associations among stem height, stem density, panicle density, grain yield, and aboveground biomass indicating that enhanced performance of contributing attributes positively affected grain yield and aboveground biomass production. A strong positive relationship between grain yield and aboveground biomass was also observed because of enhanced sink capacity, since grain productivity can be attributed to development of greater sink capacity (Zhou et al., 2019) and increased biomass productivity (Zheng et al., 2020). In contrast, planting date altered grain yield and aboveground biomass production, causing decline at PD3, possibly because of high temperature and less rainfall. Reduction in rice grain yield, as well as aboveground biomass production, was observed under reduced water supply or water stress, and prevalence of hot intervals is also well documented (Zain et al., 2014; Torres and Henry, 2018; Hussain et al., 2021b).

Reducing the gap between rice crop N requirement and N fertilization ensures enhanced efficiency of plant physiological mechanisms enabling higher N utilization plants. According to Ullah et al. (2019), the relationship between plant N uptake and N loss determines rice plant performance, and high N uptake results in increased dry matter production. Improved N management results in enhanced N uptake and utilization in plants. However, N uptake and N use in rice plants are complex mechanisms, as multiple factors, including climatic, genotypic ability to uptake N, soil properties, N volatilization, N leaching, and denitrification affect N dynamics. Increased NFR resulted in enhanced straw and grain N contents and total plant N uptake, possibly because of increased N availability. Jahan et al. (2020) noted that NFR resulted in increased rice straw and grain N contents and total plant N uptake, and the seasonal impact was significant. Planting date influenced straw and grain N contents and total plant N uptake because of high rainfall events at PD1 and low rainfall events at PD3. Nutrient availability to plant roots is linked to soil water status. We observed that PD2 received better rainfall distributions, thus the maximum straw and grain N contents and total plant N uptake. Crop water productivity indicated a significant and positive association with straw N, grain N content, and total plant N uptake. The reduced N uptake at PD3 resulted in reduced grain yield and was highly correlated with straw N, grain N contents, and total plant N uptake. Reduced source to sink activity resulted in reduced performance of upland rice. Similar results were also reported by Pal et al. (2017). Crop water productivity also indicated an increasing trend with N fertilization, and it was positively associated with plant N uptake and grain yield. An increasing trend in crop water productivity is usually accompanied by high grain yield with high N supply (Santiago-Arenas et al., 2021). However, crop water productivity was not significantly different at PD3 as compared to PD2 in both seasons in comparison to the response of other assessed attributes, particularly grain yield. Grain yield at PD2 was statistically different than at PD3, while it was statistically similar in PD1 and PD3 for N_60_ and N_90_. The contrasting trend of increase in crop water productivity was not dependent upon grain yield, as grain yield declined at PD3. Thus, this trend occurred because of decline in grain yield as well as decreased water input at PD3. NUEs, including AE_N_, RE_N_, and NHI, varied among the NFRs, while PFP decreased under increased NFR. NUE is decreased under higher N supply (Barbieri et al., 2008) in rice production systems because of high concentrations of N in the soil (Santiago-Arenas et al., 2021). Our results for PFP were in line with the finding of Santiago-Arenas et al. (2021), that the PFP and AE_N_, of direct-seeded rice decreased with increase in NFR. Variation and decline in AE_N_, RE_N_, and NHI under increased N supply possibly occurred because of increased N fertilization and low grain yield compared to control and vice versa. Thus, we observed improved N use efficiencies under N fertilization. The effect of PD differentiated the performance of N use efficiencies indicating maximum AE_N_, RE_N_, PFP, and NHI at PD2. Maximum N efficiencies at PD2 occurred because of favorable environmental conditions and improved performance of upland rice at PD2. Maximum efficiencies were reached when the NFR matched with crop N demand. Yousaf et al. (2016) also observed a similar trend for NUE in a rice and oilseed crop rotation. Crop water productivity was also associated with NUE. Furthermore, AE_N_, RE_N_, and NHI were significant and positively associated with crop water productivity, and higher crop water productivity results in enhanced NUE (Ullah et al., 2019; Lupini et al., 2021).

Our results indicate the impact of N fertilization and improvement in productivity with N fertilization according to various PDs. We have determined that the N fertilizer recommendations for upland rice production (DRRD, 2016, 2017; Norsuwan et al., 2020) and N fertilizer application rate practiced in Thailand (CARSR, 2003; Hussain et al., 2018a,b; Suwanasa et al., 2018; Islam et al., 2020) are not adequate. Another study conducted under partially controlled conditions in sheds also exhibited comparable results (Hussain et al., 2022). Hence, there is a need to adjust the current range (10–75 kg N ha^−1^) of N fertilization. A significant seasonal impact has been indicated in our findings; hence, modification of NFR as well as PD is necessary to enhance resource use efficiency. The profitability of crop production is always a concern for the farming community. In this study profitability from grain yield with N fertilization was indicated with proper PD. If the benefit-cost ratio considered fertilization with N_90_ ranked first for PD2 while considering MBCR, fertilization with N_60_ might be a suitable NFR. Thus, it should be noted that a linear relationship was indicated among agronomic traits of upland rice, N uptake and crop water productivity, and NFR. Therefore, fertilization with 90 kg N ha^−1^ is recommended for upland rice grown at PD2.

## Conclusion

Suitable N fertilizer rate (NFR) and ideal planting date (PD) increased and improved source-to-sink relationship and dry matter accumulation, which is a component for increasing the grain yield and profitability of upland rice. Agronomic adjustment in N fertilization and PD would enhance resource use efficiency. We found that N fertilization positively influenced resource use efficiency, upland rice productivity, and profitability; however, variation in PD significantly altered the results. Therefore, synchronization of NFR according to PD is necessary. We found that fertilization with 90 kg N ha^−1^ at PD2 (end of September or start of October) improved the yield and performance of yield attributes. Grain yield and crop water productivity were increased by 56 and 105% during the second and first seasons, respectively. Maximum increase in straw N, grain N content, and total plant N uptake was also observed with 90 kg N ha^−1^ for PD2 by 160, 159, and 159% during the first season, and by 153, 90, and 114%, respectively, during the second season. Variations in NUE were observed at all the NFRs in both seasons. However, maximum N efficiencies, including agronomic efficiency (AE_N_), recovery efficiency (RE_N_), partial factor productivity (PFP), and N harvest index (NHI) at varying NFRs, were observed at PD2 during both seasons. Highly significant and positive associations were found among agronomic attributes, N uptake, NUE, and crop water productivity for upland rice in a correlation assessment, indicating a direct positive impact of N fertilization. The impact of N fertilization on grain yield and profitability was observed, and application of 90 kg N ha^−1^ resulted in maximum profit at all PDs. However, the highest marginal benefit-cost ratio (MBCR) was observed with N_60_ at PD2 during both seasons. Based on the results, it was suggested that 90 kg N ha^−1^ should be applied, and that upland rice should be planted at the end of September or start of October for enhancing resource use efficiency, improving productivity, and maximum profitability. Furthermore, since a linear relationship among NFRs, agronomic traits of upland rice, N uptake, and crop water productivity was observed, and a significant seasonal effect was indicated, long-term field investigations considering a range of NFRs and adoption of forecasting measures, i.e., rainfall forecasting and yield prediction using crop simulation and modeling techniques to adjust seasonal PD, are recommended for upland rice cultivation in Thailand.

## Data Availability Statement

The original contributions presented in the study are included in the article/Supplementary Material, further inquiries can be directed to the corresponding author/s.

## Author Contributions

TH conducted and maintained the experiments and contributed to data collection and curation, investigating, methodology, visualization, formal analysis, writing the original draft, writing, reviewing, and editing the manuscript. NH conducted and maintained the experiments and contributed to data collection. HG contributed to writing, reviewing, and editing the manuscript. MA contributed to technical guidance. MT contributed to technical guidance and writing, reviewing, and editing the manuscript. SD contributed to supervision and acquiring funds, project administration, formal analysis, writing original draft, writing, reviewing, and editing the manuscript. All authors contributed to the article and approved the final version.

## Funding

This study was supported by the Thailand's Education Hub for the Southern Region of ASEAN countries' (TEH-AC) scholarship from Prince of Songkla University, Faculty of Natural Resources, Prince of Songkla University, and the CGIAR Research Program on Climate Change, Agriculture and Food Security (CCAFS) and the Global Research Alliance on Agricultural Greenhouse Gases (GRA) through their Climate, Food and Farming Global Research Alliance Development Scholarship (CLIFF-GRADS) program. CCAFS capability building objectives are carried out with support from CGIAR Trust Fund and through bilateral funding agreements. For details, please visit https://ccafs.cgiar.org/donors. Thank you to the U.S. Department of Agriculture-Agricultural Research Service for hosting the first author and to the Government of New Zealand for providing financial support. Mention of trade names or commercial products in this publication does not imply recommendation or endorsement by the U.S. Department of Agriculture-Agricultural Research Service. USDA is equal opportunity provider and employer.

## Conflict of Interest

The authors declare that the research was conducted in the absence of any commercial or financial relationships that could be construed as a potential conflict of interest.

## Publisher's Note

All claims expressed in this article are solely those of the authors and do not necessarily represent those of their affiliated organizations, or those of the publisher, the editors and the reviewers. Any product that may be evaluated in this article, or claim that may be made by its manufacturer, is not guaranteed or endorsed by the publisher.
