# Supplementary material for: Synchronizing Nitrogen Fertilization and Planting Date to Improve Resource Use Efficiency, Productivity, and Profitability of Upland Rice
Source: Front Plant Sci. 2022 May 18;13:895811. doi: 10.3389/fpls.2022.895811 (PMC9158749; doi:10.3389/fpls.2022.895811)
Supplement: Supplementary file 1 [file Data_Sheet_1.docx]

Supplementary Tables

**Supplementary Table 1.** Growing seasons soil pH, soil organic matter, total N, and available phosphorous(P) and potassium (K) at the experimental site during 2018−2019 and 2019−2020.

| Growing season | pH | Organic matter | Total N | Available P | Available K |
| --- | --- | --- | --- | --- | --- |
|  | 1:5 H_2_O, pH meter | Walkley and Black method | Kjeldahl method | Bray II method | NH_4_OAC method |
|  | − | g. kg^−1^ | g. kg^−1^ | mg. kg^−1^ | mg. kg^−1^ |
| 2018−2019 | 5.63 | 7.43 | 0.47 | 40.29 | 53.32 |
| 2019−2020 | 5.85 | 8.50 | 0.50 | 41.40 | 64.63 |

Supplementary Figures


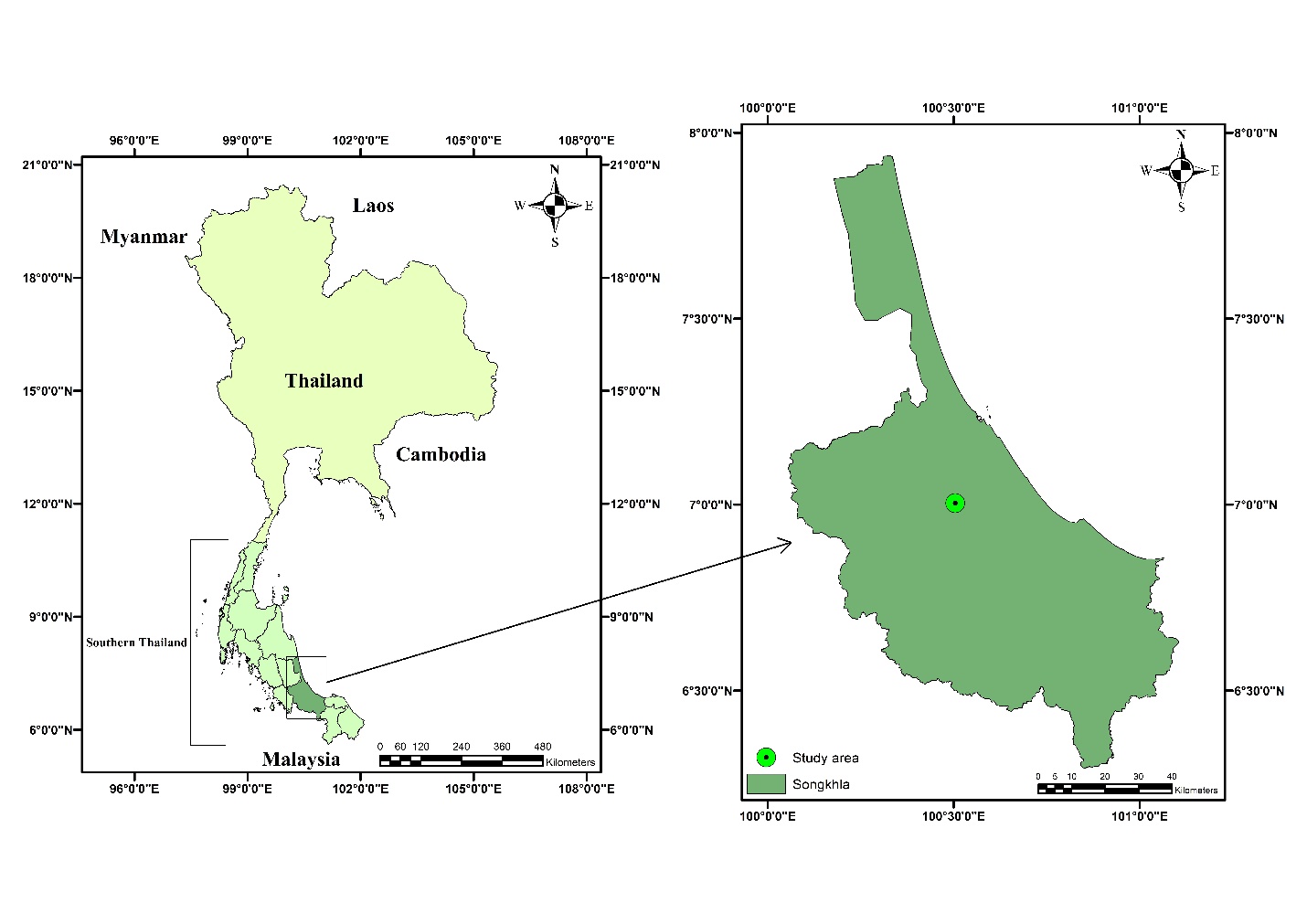


**Supplementary Figure 1.** Study area at Prince of Songkla University, Songkhla in Southern Thailand (Source: obtained from ArcGIS software: *version*−10.5).
